# Linking the Dynamic Response of the Carbon Dioxide-Concentrating Mechanism to Carbon Assimilation Behavior in Fremyella diplosiphon

**DOI:** 10.1128/mBio.01052-20

**Published:** 2020-05-26

**Authors:** Brandon A. Rohnke, Kiara J. Rodríguez Pérez, Beronda L. Montgomery

**Affiliations:** a DOE—Plant Research Laboratory, Michigan State University, East Lansing, Michigan, USA; b Department of Biochemistry and Molecular Biology, Michigan State University, East Lansing, Michigan, USA; c University of Puerto Rico at Arecibo, Arecibo, Puerto Rico; d Department of Microbiology and Molecular Genetics, Michigan State University, East Lansing, Michigan, USA; University of Washington

**Keywords:** carbon dioxide assimilation, carbon dioxide concentration mechanism, carbon dioxide fixation, carboxysome, cyanobacteria, gas exchange

## Abstract

Cyanobacteria use a carbon dioxide (CO_2_)-concentrating mechanism (CCM) that enhances their carbon fixation efficiency and is regulated by many environmental factors that impact photosynthesis, including carbon availability, light levels, and nutrient access. Efforts to connect the regulation of the CCM by these factors to functional effects on carbon assimilation rates have been complicated by the aqueous nature of cyanobacteria. Here, we describe the use of cyanobacteria in a semiwet state on glass fiber filtration discs—cyanobacterial discs—to establish dynamic carbon assimilation behavior using gas exchange analysis. In combination with quantitative PCR (qPCR) and transmission electron microscopy (TEM) analyses, we linked the regulation of CCM components to corresponding carbon assimilation behavior in the freshwater, filamentous cyanobacterium Fremyella diplosiphon. Inorganic carbon (C_i_) levels, light quantity, and light quality have all been shown to influence carbon assimilation behavior in F. diplosiphon. Our results suggest a biphasic model of cyanobacterial carbon fixation. While behavior at low levels of CO_2_ is driven mainly by the C_i_ uptake ability of the cyanobacterium, at higher CO_2_ levels, carbon assimilation behavior is multifaceted and depends on C_i_ availability, carboxysome morphology, linear electron flow, and cell shape. Carbon response curves (CRCs) generated via gas exchange analysis enable rapid examination of CO_2_ assimilation behavior in cyanobacteria and can be used for cells grown under distinct conditions to provide insight into how CO_2_ assimilation correlates with the regulation of critical cellular functions, such as the environmental control of the CCM and downstream photosynthetic capacity.

## Abstract (precis)

Environmental regulation of photosynthesis in cyanobacteria enhances organismal fitness, light capture, and associated carbon fixation under dynamic conditions. Concentration of carbon dioxide (CO_2_) near the carbon-fixing enzyme RubisCO occurs via the CO_2_-concentrating mechanism (CCM). The CCM is also tuned in response to carbon availability, light quality or levels, or nutrient access—cues that also impact photosynthesis. We adapted dynamic gas exchange methods generally used with plants to investigate environmental regulation of the CCM and carbon fixation capacity using glass fiber-filtered cells of the cyanobacterium Fremyella diplosiphon. We describe a breakthrough in measuring real-time carbon uptake and associated assimilation capacity for cells grown in distinct conditions (i.e., light quality, light quantity, or carbon status). These measurements demonstrate that the CCM modulates carbon uptake and assimilation under low-C_i_ conditions and that light-dependent regulation of pigmentation, cell shape, and downstream stages of carbon fixation are critical for tuning carbon uptake and assimilation.

## INTRODUCTION

**FIG 1:**
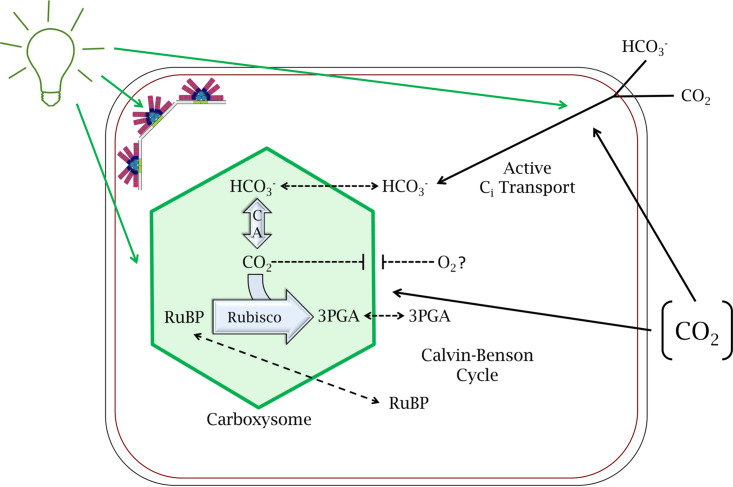
Generalized schematic of the carbon-concentrating mechanism (CCM) in a cyanobacterial cell. The CCM is comprised of the flux of C_i_ (as both HCO_3_- and CO_2_) into a cyanobacterial cell and the carboxysome, a proteinaceous microcompartment which contains RubisCO. This flux of C_i_ and the CCM are regulated and tuned at many points, including by light availability and by the concentration of available CO_2_ in the external environment. Light quality and quantity tune multiple aspects associated with CCM and carbon fixation (represented by solid green lines), including tuning phycobilisomes (which are represented by the colored fan-like structures, including the core and rods of hemidiscoidal phycobilisomes typical of Fremyella diplosiphon) that impact the overall efficiency of light harvesting associated with carbon fixation, carboxysome dynamics (size and number per cell), and carbon transporters. Carbon dioxide availability also can impact carbon transporter abundance and carboxysome dynamics (represented by solid black arrows extending from [CO_2_]).

The robust capability of cyanobacteria to fix carbon through photosynthesis is critical to their ecological role as one of Earth’s major primary producers. Cyanobacteria concentrate inorganic carbon (C_i_) through a well-established CO_2_-concentrating mechanism (CCM) (see the review in reference 1), which sequesters carbon dioxide and related enzymes and substrates in subcellular, proteinaceous bacterial microcompartments (BMCs) called carboxysomes (see the review in reference 2). As the carbon fixation steps of photosynthesis are often regulated to ensure that they are kept in balance with the overall rate of photosynthesis (3), components of the CCM are likely to be tuned to environmental factors that affect photosynthesis, as well (Fig. 1). Indeed, both carbon transport and carboxysome components are upregulated under conditions where there is a greater need for C_i_ uptake and fixation, such as during growth under conditions of low CO_2_ or high light (HL) (4, 5). We are interested in the specific means by which cyanobacteria regulate modular components of the CCM, such as the carbon transporters and carboxysome dynamics, to coordinately control the rate of photosynthesis and associated cellular fitness.

The CCM has two main functions: C_i_ uptake and C_i_ fixation. C_i_ uptake is the first step of the CCM. Since the cellular membrane is permeable to CO_2_ but not HCO_3_-, cyanobacteria increase the flux of C_i_ into the cell using HCO_3_- transporters and trap CO_2_ as HCO_3_- using CO_2_-hydrating enzymes. Constitutive active carbon transport (4, 6) involves the low-affinity Na^+^/HCO_3_- symporter Bic in the cellular membrane (7) and the hydration of cytosolic CO_2_ into HCO_3_- by membrane-localized NDH-1_4_ (including subunits D4/F4/CupB) (8, 9). Together, these components drive HCO_3_- accumulation inside the cell. A parallel set of proteins with higher substrate affinity can be induced to increase C_i_ uptake and includes SbtA, an inducible Na^+^/HCO_3_- symporter (10); BCT1, an ATP-dependent HCO_3_- pump (11); and NDH-1_3_ (subunits D3/F3/CupA) at the thylakoid membrane (6, 8, 12). These complexes provide cyanobacteria with a high and tunable capacity for regulating internal C_i_-influx as HCO_3_-.

The second step of the CCM, C_i_ fixation, occurs in the carboxysome, which is a subcellular compartment with a proteinaceous shell that is permeable to HCO_3_- but not CO_2_ (13). Both RubisCO and carbonic anhydrase (CA) are part of the carboxysomal cargo and, in conjunction with the high concentration of cellular HCO_3_-, drive the carboxylation reaction of RubisCO forward with high local concentrations of its CO_2_ substrate. In the case of β-carboxysomes, which represent the type of carboxysomes formed in organisms such as Fremyella diplosiphon with type 1B RubisCO, the *ccmKMNO* operon is vital to carboxysome formation (14). Biogenesis of β-carboxysomes begins with RubisCO aggregation by CcmM (15), a protein that can interact with L_8_S_8_ RubisCO (16, 17). CcmN is then recruited to this condensate, and, alongside full-length CcmM, interacts with CcmK2, the most abundant shell protein, at a minimum (15, 18, 19). Other shell protein paralogs that may also be found in carboxysomes include CcmK1, CcmK3, CcmK4, CcmK5, CcmK6, CcmO, CcmP, and CcmL (15, 20–26).

The CCM found in cyanobacteria has multiple modular components that can respond to dynamic environmental conditions and impact photosynthetic capacity in diverse habitats. Both HL and low CO_2_ levels tend to induce the expression of genes encoding many CCM components, especially for high-affinity carbon transporters (4, 27, 28). It has also been demonstrated that carboxysome morphology is dynamically responsive to light, C_i_ availability and concentration, and the photosensory activity of cyanobacteriochromes, including regulation of expression of carboxysome structural genes (5, 29, 30). However, many questions remain with respect to understanding how these environmentally tuned changes control the carbon fixation capability of cyanobacteria.

Given these known biological responses, there has been an effort to cohesively model how the complex photosynthetic parameters of cyanobacteria arise from regulation of the CCM (30–33). These efforts are generally limited to single-celled model cyanobacteria and are often inadequate for quickly measuring net C_i_ consumption due to the aqueous nature of these organisms. Several distinct methods for assaying carbon uptake, fixation, and overall photosynthesis have been applied to cyanobacteria. It is perhaps most common to measure O_2_ evolution, which probes linear electron flow at photosystem II (PSII) and shows reductions when CCM is compromised (34–36). Chlorophyll (Chl) fluorescence similarly can be used but requires care in cyanobacteria to avoid interference from phycobilisome absorbance or fluorescence (37). Carbon labeling also has utility for determining rates of carbon assimilation and flux. Due to the equilibration between CO_2_ and HCO_3_-, both the media and cytosol can have stores of C_i_ that are separate from what is fixed, so care must be taken to distinguish between stores and the assimilation of CO_2_ and HCO_3_- (33, 38, 39). In general, the aforementioned measurements are limited to endpoint assays and/or are technically challenging.

For terrestrial plants, a robust method derives net gas exchange from a plot of carbon assimilation versus intracellular CO_2_ to establish steady-state photosynthetic parameters nondestructively (40). Carbon assimilation versus intracellular CO_2_ curves from plants are typically modeled with three distinct regions: at low levels of intercellular C_i_ assimilation, rates are limited by the reaction rate of RubisCO; at higher levels of intercellular C_i_ assimilation, rates are limited by the rate of ribulose-1,5-bisphosphate regeneration (light-limited); and at the highest intercellular C_i_ values, the assimilation curves may show saturation due to maximum utilization of triose phosphate pools (41). Due to the aqueous nature of cyanobacteria and the slow, uncatalyzed equilibration of HCO_3_- with CO_2_, parallel methods have yet to be well established but those that have been examined are promising (32, 33). Notably, Douchi et al. recently demonstrated that the response to declining C_i_ can be modeled with a two-phase sigmoidal curve in *Synechocystis* sp. PCC 6803 (here referred to as *Synechocystis*) (33), reminiscent of the carbon assimilation versus intracellular CO_2_ curves seen in C_4_ plants (42, 43). Their work supports a biphasic model that indicates rate limitations imposed by the CCM for the lower phase and by the Calvin-Benson cycle (represented by a C_i_ fixation coefficient) for the upper phase. This biphasic model offers a framework for modeling carbon fixation more broadly in cyanobacteria.

In this study, we analyzed the carbon fixation characteristics of F. diplosiphon, which exhibits complementary chromatic acclimation (CCA). CCA is a process whereby cells respond to changes in the prevalence of light (primarily red versus green in F. diplosiphon and many other cyanobacteria) by altering the type and abundance of photosynthetic pigments, cell shape, and filament length (44, 45). Notably, cyanobacteriochrome RcaE acts as a photoreceptor that controls CCA (46–49) and contributes to the photoregulation of carboxysome morphology (29). Given the role of RcaE in regulating dynamic organismal responses to light, we hypothesized that this photoreceptor may serve to coordinate critical aspects of cells’ dynamic regulation of carbon assimilation and associated organismal fitness. In order to investigate the roles of CCA and CCM regulation in tuning carbon assimilation (e.g., the net rate of CO_2_ uptake per unit area), we demonstrate that carbon assimilation can be measured progressively using cyanobacteria in a semiwet state with infrared gas analysis of cyanobacterial discs. We investigated the impact of dynamic environmental factors, including light (quality and quantity), C_i_ availability, and the physiological state of cells during carbon assimilation, on wild-type (WT) F. diplosiphon and a number of mutant strains. We show that dynamic responses of carbon assimilation can be evaluated using carbon response curves (CRCs) in cyanobacteria and, together with measurements such as O_2_ evolution, can be used to infer the propensity of cells to exhibit C_i_ uptake and active utilization during oxygenic photosynthesis.

## RESULTS

### Carbon assimilation measurements of responses of F. diplosiphon to light, inorganic carbon availability, and physiological state.

**FIG 2:**
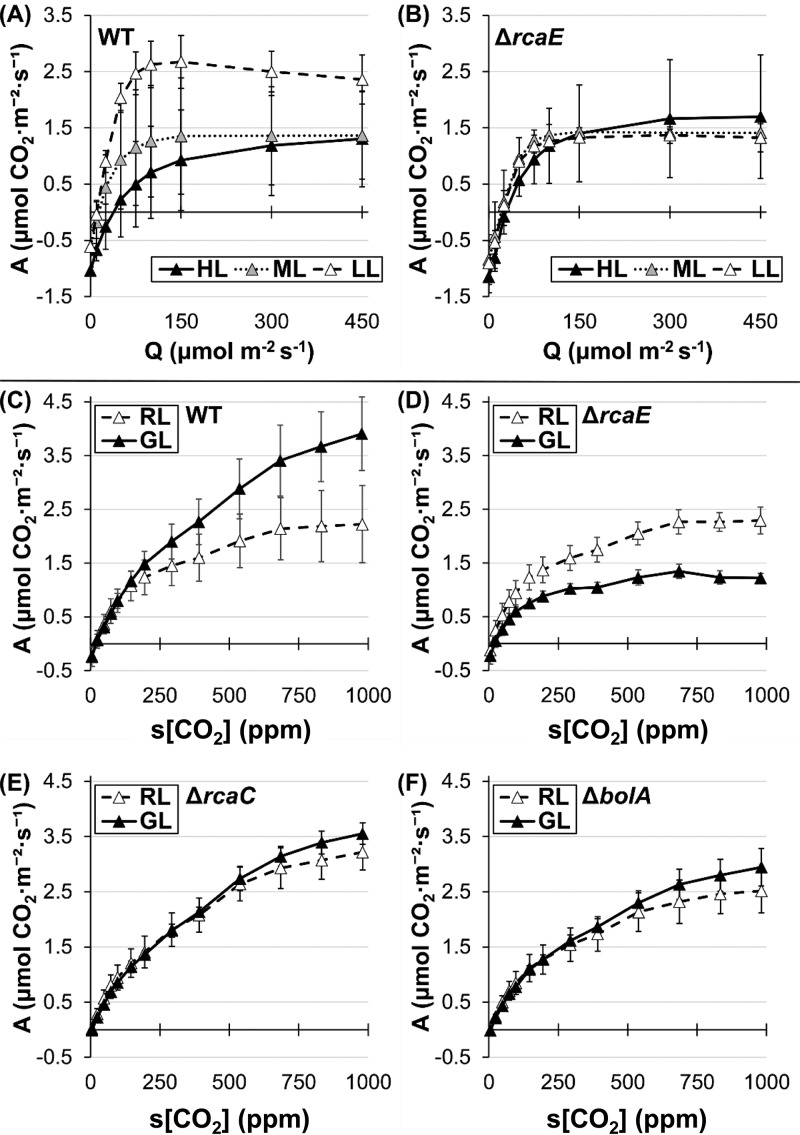
Carbon assimilation response to light and C_i_ availability. (A and B) Carbon assimilation (“A”) response (expressed in μmol m^−2^ s^−1^) to Li-COR chamber light at 400 ppm s[CO_2_] for WT (A) and Δ*rcaE* (B) F. diplosiphon strains grown at low (12 μmol·m^−2^·s^−1^; white symbols), medium (30 μmol·m^−2^·s^−1^; gray symbols), and high (100 μmol·m^−2^·s^−1^; black symbols) WL intensity in air. *n* = 3 for LL and the Δ*rcaE* mutant ML, and *n* = 5 for HL and WT ML. (C to F) Carbon assimilation (“A”) response (expressed in μmol m^−2^ s^−1^) to CO_2_ supplied at 300 μmol·m^−2^·s^−1^ for WT (C), the Δ*rcaE* mutant (D), the Δ*rcaC* mutant (E), and Δ*bolA* (F) F. diplosiphon strains grown under ∼10 to 12 μmol·m^−2^·s^−1^ RL (white symbols) or GL (black symbols) conditions. Error bars represent 95% confidence intervals for *n* ≥ 3 from 2 independent biological replicates.

Glass fiber-filtered F. diplosiphon strains (i.e., F. diplosiphon discs) were analyzed in a semiwet state with infrared gas analysis to detect CO_2_ uptake and consumption. Carbon assimilation rates in WT and Δ*rcaE* F. diplosiphon strains were responsive to light intensity, showing light saturation at ∼100 μmol·m^−2^·s^−1^ and ∼300 μmol·m^−2^·s^−1^ in low-light (LL) and HL-acclimated cultures, respectively (Fig. 2A and B). Thus, 300 μmol·m^−2^·s^−1^ was selected for saturating light in further experiments. Under these conditions, strains of F. diplosiphon exhibited changes in carbon assimilation in response to changing carbon levels in a standard CRC (Fig. 2C to F). Blank glass fiber-filtered discs wetted with fresh cell media were used as a control and showed slightly negative assimilation values that became more negative from 600 to 1,000 ppm (see Fig. S1 in the supplemental material). Samples were normalized by optical density at 750 nm (OD_750_), which had a roughly linear relationship with [Chl*a*] (Fig. S2). As the intercellular C_i_ flux in cyanobacteria is complex and has not been modeled precisely, response curves are presented with the [CO_2_] levels in the sample chamber (s[CO_2_]) as the independent variable. As in plants, these CRCs follow a generally sigmoidal curve and are expected to be limited by C_i_ availability at low C_i_ values and by other factors such as light availability when C_i_ levels are saturating. Compensation points (near the point where assimilation becomes negative and which represent equivalent rates of photosynthetic CO_2_ flux and respiration) appear to fall between 5 and 25 ppm s[CO_2_] in cyanobacterial CRCs, which are likely lower than the typical values (25 to 100 ppm intercellular CO_2_) found in plants (41, 50). These observations are consistent with the presence of a CCM in cyanobacteria.

10.1128/mBio.01052-20.1 FIG S1 Carbon assimilation response to C_i_ availability for BG11/HEPES blank. Data represent carbon assimilation (“A”) responses (expressed in μmol m^−2^ s^−1^) to CO_2_ supplied at 300 μmol·m^−2^·s^−1^ for BG11/HEPES filtered through Whatman glass fiber filter paper as a blank for the semiwet cyanobacteria gas exchange analysis. Error bars represent a 95% confidence interval from 3 replicates. Download FIG S1, TIF file, 0.1 MB. Copyright © 2020 Rohnke et al. 2020 Rohnke et al. This content is distributed under the terms of the Creative Commons Attribution 4.0 International license.

10.1128/mBio.01052-20.2 FIG S2 Chlorophyll *a* levels versus OD_750_ for cyanobacteria used in CRC analysis. Data are representative of [Chl*a*] versus OD_750_ measured in extracts harvested during CRC runs. Samples include WT (A) and Δ*rcaE* (B) F. diplosiphon strains grown under red light (RL), green light (GL), low light (LL), medium light (ML), or high light (HL) WL conditions or in RL-enriched WL under air (Air), C_i_ upshift (C_i_ Up), or C_i_ downshift (C_i_ Down) conditions as described in Materials and Methods. Download FIG S2, TIF file, 0.1 MB. Copyright © 2020 Rohnke et al. 2020 Rohnke et al. This content is distributed under the terms of the Creative Commons Attribution 4.0 International license.

This CRC method was then used to compare cultures acclimated to red light (RL) and green light (GL). The WT strain showed significant differences in carbon assimilation only above 700 ppm CO_2_, i.e., beyond the C_i_-limited region of the CRCs, with GL-grown cultures reaching higher assimilation levels (Fig. 2C). This result is consistent with previous measurements of O_2_ evolution, which revealed similar rates of O_2_ evolution for F. diplosiphon grown in low-intensity RL compared to GL at ambient CO_2_, which would correspond to the C_i_-limited region (37). The Δ*rcaE* mutant, which has more numerous and smaller carboxysomes than the WT in both RL and GL (29), demonstrated impeded carbon assimilation only under GL conditions. The maximum assimilation rate dropped from ∼4.0 for the WT to ∼1.3 μmol·m^−2^·s^−1^ for the Δ*rcaE* mutant in GL. By comparison, the assimilation rate seen with the Δ*rcaE* mutant was statistically indistinguishable from that seen with the WT under RL conditions (Fig. 2C and D).

We hypothesized that differences in cellular pigmentation in the WT under RL versus GL conditions contribute to light-dependent differences in the net rate of CO_2_ uptake. Thus, we measured the carbon assimilation rate in a Δ*rcaC* mutant strain with constitutively GL-like pigmentation (51), due to the lack of the DNA-binding regulatory protein RcaC, which acts downstream of RcaE. CRC analysis indicated no differences in the assimilation values for the Δ*rcaC* strain between RL and GL, with values more similar to the WT values seen under GL conditions (Fig. 2E). This finding suggests that the GL physiological state is partially responsible for the higher assimilation values measured under conditions that employed that light quality in the WT.

In addition to pigmentation differences, WT F. diplosiphon exhibits cell shape differences that are controlled in part by RcaE, with spherical cells in RL and rod-shaped cells in GL (46). We hypothesized that cell shape and its regulation contribute to light-dependent differences in measured carbon assimilation rates, perhaps due to differences in gas diffusion levels in spherical cells compared to rod-shaped cells. Thus, we analyzed carbon assimilation in a Δ*bolA* mutant strain with an altered, constitutively more spherical cell shape (48). As the strain had WT pigmentation, analysis of the Δ*bolA* mutant relative to the WT allowed us to separate the potential impacts of pigmentation regulation from the regulation of cell shape. Assimilation values in the Δ*bolA* mutant showed no differences between RL and GL and were closer to the assimilation values for the WT under RL conditions (Fig. 2F). Since assimilation in the Δ*bolA* mutant was similar to that measured for spherical WT cells in RL, the regulation of cell shape likely plays a role in CRC behavior whereas pigmentation does not appear to have a significant role.

### Effect of nonsaturating light on carbon assimilation.

**FIG 3:**
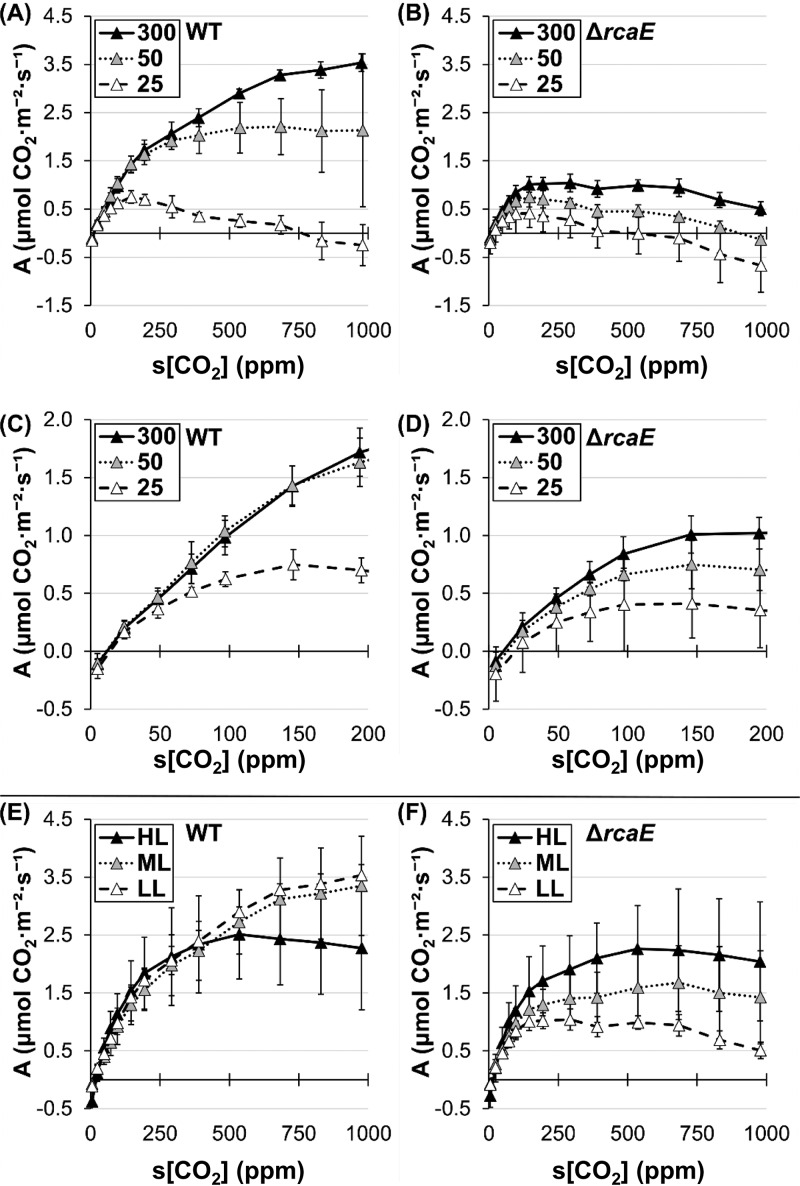
Carbon assimilation response to C_i_ availability in response to various light intensities. (A to D) Carbon assimilation (“A”) response (expressed in μmol m^−2^ s^−1^) to CO_2_ supplied during runs at 300 μmol·m^−2^·s^−1^ (black symbols), 50 μmol·m^−2^·s^−1^ (gray symbols), or 25 μmol·m^−2^·s^−1^ (white symbols) for WT (A and C) and Δ*rcaE* (B and D) F. diplosiphon strains grown at low (12 μmol·m^−2^·s^−1^) GL-enriched WL. Panels C and D show data corresponding to 0 to 200 ppm s[CO_2_] in panels A and B, respectively. Error bars represent 95% confidence intervals for *n* ≥ 3 from 2 independent biological replicates. (E and F) Carbon assimilation (“A”) response (expressed in μmol m^−2^ s^−1^) to CO_2_ supplied at 300 μmol·m^−2^·s^−1^ for WT (E) and Δ*rcaE* (F) F. diplosiphon strains grown at low (12 μmol·m^−2^·s^−1^; white symbols), medium (30 μmol·m^−2^·s^−1^; gray symbols), and high (100 μmol·m^−2^·s^−1^; black symbols) GL-enriched WL intensities in air. Error bars represent 95% confidence intervals for *n* ≥ 4 from 2 independent biological replicates.

In order to probe for the light-limited regions of the CRC in cyanobacteria, we performed analyses under nonsaturating test light conditions, i.e., using 25 and 50 μmol·m^−2^·s^−1^ of light compared to the prior parameters of 300 μmol·m^−2^·s^−1^. WT F. diplosiphon grown under LL conditions had near-saturation carbon assimilation values, even at light measurements as low as 50 μmol·m^−2^·s^−1^ (Fig. 3A). However, assimilation was severely impaired 25 μmol·m^−2^·s^−1^ above 75 ppm s[CO_2_] (Fig. 3C). The level of assimilation exhibited by the Δ*rcaE* mutant was also decreased under nonsaturating light conditions and was indistinguishable from the WT level at 25 μmol·m^−2^·s^−1^ (Fig. 3B and D).

### Effect of different light intensities during growth on carbon assimilation potential.

Since HL is known to induce the components of CCM (4, 5, 27), we hypothesized that growth of F. diplosiphon under conditions of increasing light intensity would support higher assimilation values via induction of C_i_ uptake and increased linear electron flow until the levels of light that were reached were stressful or induced phototoxicity. We used a multicultivator bioreactor system with green-enriched white light (WL) at LL (12 μmol·m^−2^·s^−1^), medium light (ML; 30 μmol·m^−2^·s^−1^), or HL (100 μmol·m^−2^·s^−1^) intensities to measure assimilation rates in the WT and the Δ*rcaE* mutant. Although the growth rate increased as light intensity increased in both strains (Fig. S3), cells typically exhibited chlorosis at ∼7 days after induction of HL, indicating light stress. CRC analysis of the WT indicated that the responses to LL and ML were similar. HL caused a general decreasing trend in CO_2_ assimilation levels at high s[CO_2_] in the WT, with substantial variation, but with assimilation levels significantly lower than those seen under LL or ML conditions at s[CO_2_] levels of ≥700 ppm (Fig. 3E). In contrast, we observed a general increase in assimilation rates in the Δ*rcaE* mutant during growth under conditions of increasing light intensity, with assimilation approaching near-WT levels under HL conditions and with significant differences between the levels of CO_2_ assimilation for LL compared to HL at higher s[CO2] levels (Fig. 3E and F). In addition, under conditions of HL acclimation, the two strains exhibited low, indistinguishable assimilation values under nonsaturating light conditions (Fig. S4).

10.1128/mBio.01052-20.3 FIG S3 Growth rates of F. diplosiphon strains under conditions of increasing GL-enriched WL intensity. OD_720_ values versus time are shown for WT (A) and Δ*rcaE* (B) F. diplosiphon strains grown under WL with dominant GL wavelengths at low (12 μmol·m^−2^·s^−1^; black lines), medium (30 μmol·m^−2^·s^−1^; purple lines), or high (100 μmol·m^−2^·s^−1^; blue lines) intensity. The shaded area represents ± SD for *n* ≥ 4 from at least 2 independent biological replicates. Download FIG S3, TIF file, 0.2 MB. Copyright © 2020 Rohnke et al. 2020 Rohnke et al. This content is distributed under the terms of the Creative Commons Attribution 4.0 International license.

10.1128/mBio.01052-20.4 FIG S4 Carbon assimilation response to C_i_ availability under nonsaturating light conditions after acclimation to HL. Data represent carbon assimilation (“A”) responses (expressed in μmol m^−2^ s^−1^) to CO_2_ supplied at 300 μmol·m^−2^·s^−1^ (black symbols) or 25 μmol·m^−2^·s^−1^ (white symbols) for WT (A and C) and Δ*rcaE* (B and D) F. diplosiphon strains grown at high (100 μmol·m^−2^·s^−1^) WL intensity. Panels C and D show 0 to 200 ppm s[CO_2_] corresponding to panels A and B, respectively. Error bars represent 95% confidence intervals for *n* ≥ 3 from 2 independent biological replicates. Download FIG S4, TIF file, 0.3 MB. Copyright © 2020 Rohnke et al. 2020 Rohnke et al. This content is distributed under the terms of the Creative Commons Attribution 4.0 International license.

### Effect of inorganic carbon availability on carbon assimilation during growth.

**FIG 4:**
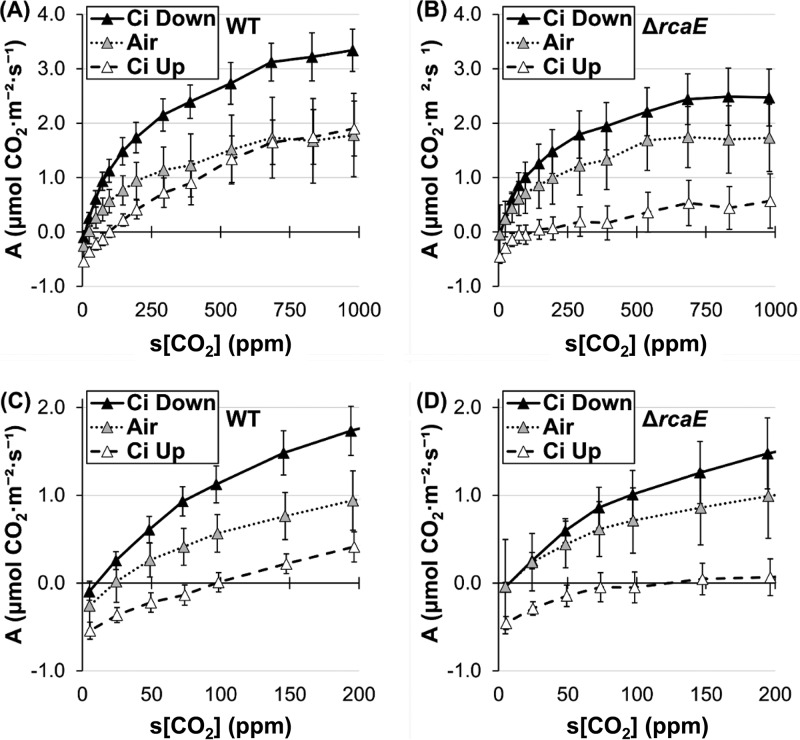
Carbon assimilation response to C_i_ availability after acclimation to various C_i_ levels. Data represent carbon assimilation (“A”) response (expressed in μmol m^−2^ s^−1^) to CO_2_ supplied at 300 μmol·m^−2^·s^−1^ for WT (A and C) and (B and D) Δ*rcaE* F. diplosiphon strains grown at medium (∼35 μmol·m^−2^·s^−1^) RL-enriched WL intensity in air with 3% CO_2_ enrichment (Ci Up; black symbols), without enrichment (Air; gray symbols), or under conditions of C_i_ downshift (Ci Down; white symbols). Panels C and D show data corresponding to 0 to 200 ppm s[CO_2_] in panels A and B, respectively. Error bars represent 95% confidence intervals for *n* ≥ 4 from 2 independent biological replicates.

**FIG 5:**
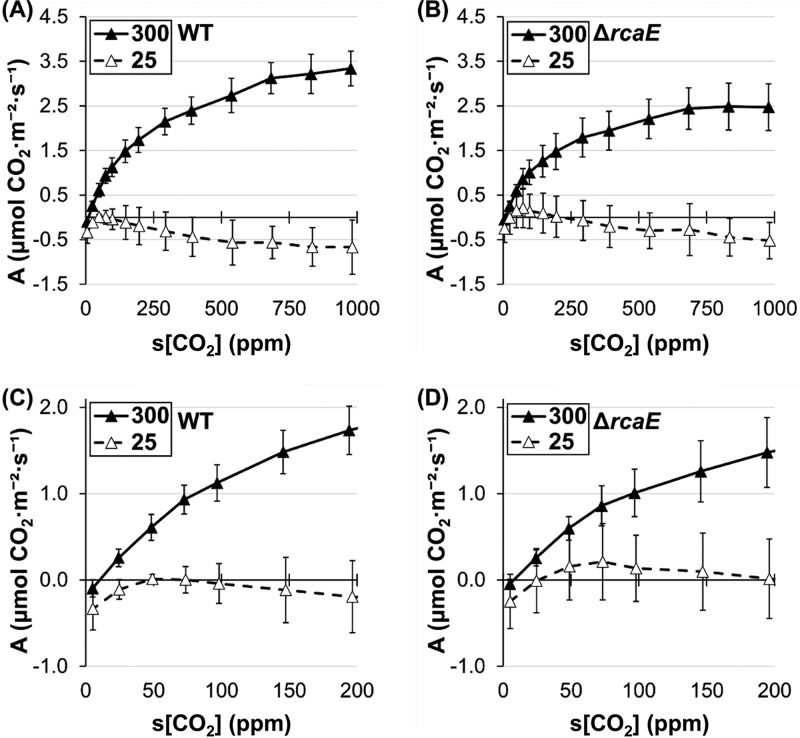
Carbon assimilation response to C_i_ availability in nonsaturating light after acclimation to C_i_ downshift. Data represent carbon assimilation (“A”) response (expressed in μmol m^−2^ s^−1^) to CO_2_ supplied at 300 μmol·m^−2^·s^−1^ (black symbols) or 25 μmol·m^−2^·s^−1^ (white symbols) for WT (A and C) and Δ*rcaE* (B and D) F. diplosiphon strains grown at medium (∼35 μmol·m^−2^·s^−1^) RL-enriched WL intensity under conditions of C_i_ downshift. Panels C and D show data corresponding to 0 to 200 ppm s[CO_2_] in panels A and B, respectively. Error bars represent 95% confidence intervals for *n* ≥ 3 from 2 independent biological replicates.

We next explored the impact of C_i_ availability on CRC behavior. Cells were grown in air or under conditions of C_i_ upshift (3% CO_2_) or C_i_ downshift (3 days growth in 3% CO_2_ followed by a transfer to air for 19 h) in chambers illuminated with 35 to 40 μmol·m^−2^·s^−1^ WL (Fig. 4). The WT and Δ*rcaE* strains exhibited similar carbon assimilation behaviors under conditions of exposure to air (Fig. 4A and B). The behaviors of these two strains were similar at below 200 ppm s[CO_2_] under all conditions, and, as expected, the compensation point appeared to decrease as the cultures became more acclimated to lower C_i_ levels and induced high-affinity CCM systems (Fig. 4C and D). During acclimation to C_i_ downshift, the two strains also performed similarly to each other in runs under nonsaturating light conditions (Fig. 5). However, the Δ*rcaE* mutant strain exhibited a deficiency in response to C_i_ levels with reduced assimilation under conditions of C_i_ upshift and a less robust response to C_i_ downshift than the WT at higher s[CO_2_] levels (Fig. 4B).

### Rates of O_2_ evolution in F. diplosiphon strains under RL and GL conditions.

**FIG 6:**
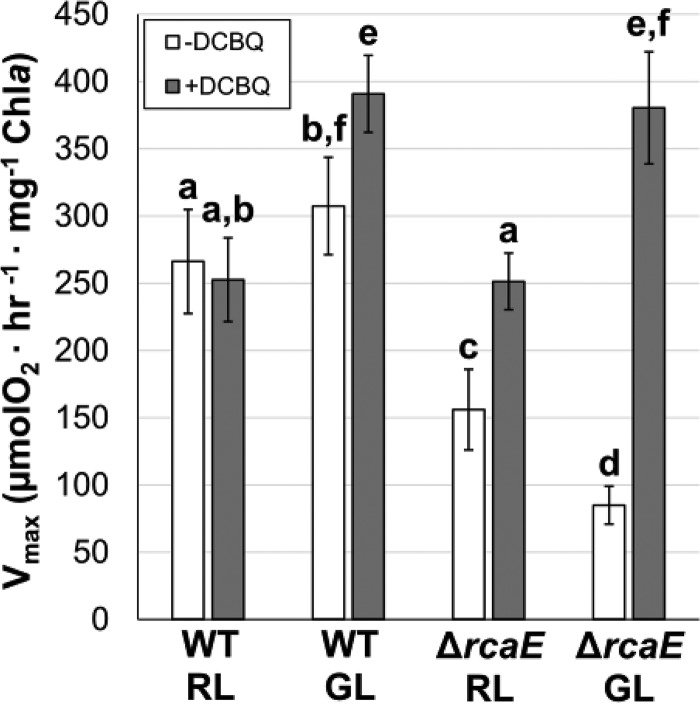
Oxygen evolution of F. diplosiphon strains acclimated to RL or GL. Data represent O_2_ levels measured after illumination by 250 μmol·m^−2^·s^−1^ WL in F. diplosiphon strains (WT or the Δ*rcaE* mutant) grown under RL or GL conditions, with or without the addition of a 0.2 mM concentration of the electron acceptor DCBQ and 1.5 mM FeCN. Error bars represent standard deviations for *n* = 10 (−DCBQ) or *n* = 3 (+DCBQ) from ≥ 2 independent biological replicates. Lowercase letters indicate statistically significant groups (*P* < 0.05) determined using a Student's *t* test.

To compare our findings to those obtained using established methods and to compare CO_2_ uptake with active C_i_ utilization in oxygenic photosynthesis, we analyzed O_2_ evolution in WT and Δ*rcaE* strains that had been acclimated to RL or GL (Fig. 6, white bars). The WT produced O_2_ at marginally higher initial rates in GL than were seen with cells grown in RL (*P* = 0.024). O_2_ evolution was significantly decreased in the Δ*rcaE* mutant relative to the WT under both RL and GL conditions. Whereas CRC analysis uncovered a defect in carbon assimilation only under GL conditions, the Δ*rcaE* strain showed reduced O_2_ evolution rates compared to the WT even after acclimation to RL. We treated cells with 2,6-dichloro-*p*-benzoquinone (DCBQ; 0.2 mM), which accepts electrons from PSII and enables tests to determine the total number of PSII centers capable of water oxidation (52, 53). The WT exhibited similar levels of O_2_ evolution in RL with or without DCBQ but exhibited higher O_2_ evolution levels in GL after DCBQ was added (Fig. 6). The latter response for the WT was anticipated as the addition of 0.5 mM DCBQ in *Synechocystis* was previously shown to increase O_2_ evolution rates substantially (53). The fact that the rates did not increase in WT F. diplosiphon in RL suggests that this strain utilizes the majority of its PSII complexes that have sufficient excitement to split water (i.e., downstream regulation does not limit the WT in RL) under this light condition. However, in GL, cell activity may be limited by downstream reactions. Furthermore, the decrease of carbon assimilation rates seen under RL compared to GL conditions (Fig. 2C) may be attributable to the PSII reaction rates, as the WT under RL conditions exhibited lower O_2_ evolution rates with and without DCBQ compared to cognate samples in GL. O_2_ evolution rates increased in DCBQ-treated Δ*rcaE* cultures in both RL and GL (Fig. 6). However, the Δ*rcaE* mutant showed no significant differences from the WT under either light condition for DCBQ-treated cultures. This finding suggests that the apparent reduction in the photosynthetic rate of the Δ*rcaE* mutant under GL conditions (as measured by both carbon assimilation and O_2_ evolution rates) is not due to a deficiency in PSII reaction rates but might be associated with aspects of carbon utilization.

### Transmission electron microscopy (TEM) analysis of carboxysome morphology in response to light conditions and carbon availability.

**FIG 7:**
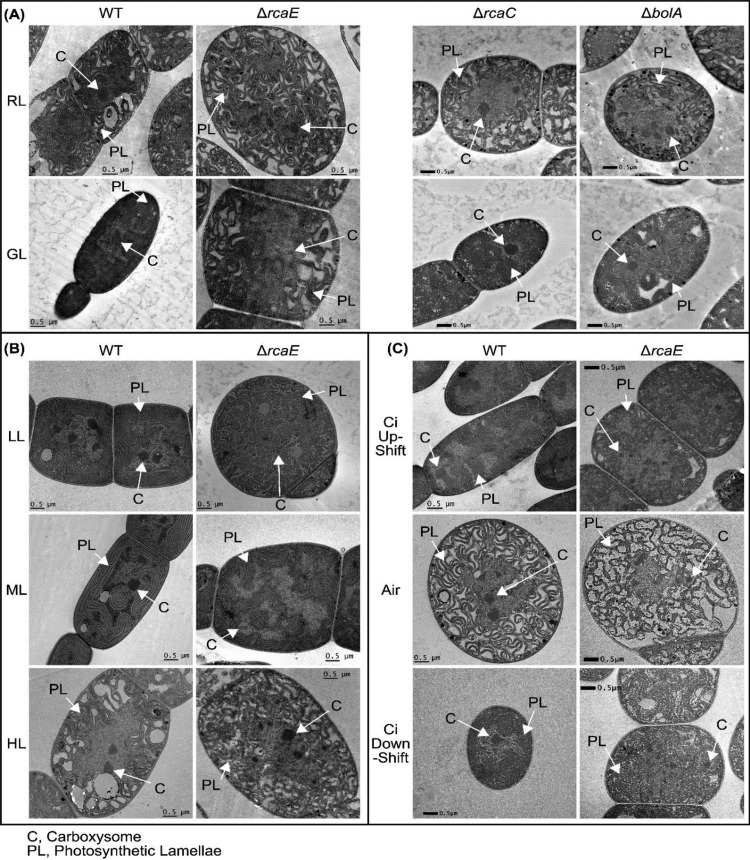
TEM analysis of cellular morphology of F. diplosiphon strains under conditions of changing light or C_i_ availability. Images are representative of WT, Δ*rcaE*, Δ*rcaC*, and Δ*bolA* strains grown under RL and GL conditions (A) and of WT and Δ*rcaE* strains grown under conditions of increasing WL intensity (B) or altered CO_2_ availability (C). Bars, 0.5 μm. C, carboxysomes; PL, photosynthetic lamellae.

**FIG 8:**
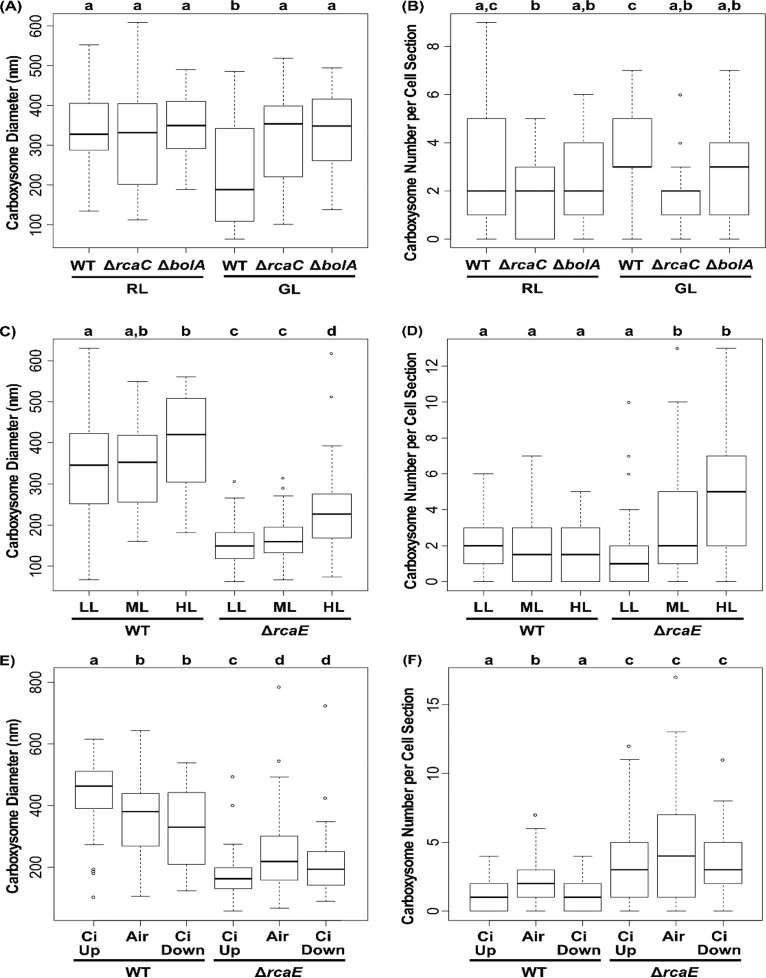
Carboxysome morphology under diverse physiological conditions. Box plots display the full range of measurements of maximum carboxysome diameter and the number of carboxysomes per cell section from the TEM analysis for the WT, Δ*rcaC*, and Δ*bolA* strains of F. diplosiphon grown under RL and GL conditions (A and B) and the WT and Δ*rcaE* strains grown under conditions of increasing WL intensity (C and D) or altered CO_2_ availability (E and F). Lowercase letters indicate statistically significant groups (*P* < 0.05) within a panel, obtained using a Student's *t* test. The corresponding averages ± standard errors (SE) and sample sizes are presented in Table 1. Data for the WT strain grown under RL and GL conditions are reproduced here from a study previously published by Rohnke et al. (29) under the terms of the Creative Commons Attribution 4.0 International license, and data for the WT strain grown under conditions of air and C_i_ upshift are reproduced here from a study previously published by Lechno-Yossef et al. (54) with permission from the publisher.

To contextualize the CRC behaviors and investigate which may be associated with a specific carboxysome morphology, we analyzed carboxysome dynamics under the conditions used for CRC analyses (Fig. 7). In addition to the altered carboxysome size and number in the Δ*rcaE* mutant compared to the WT in both RL and GL (29), the diameter of carboxysomes decreased in both strains under GL conditions and there were no light quality-dependent changes in carboxysome abundance in either strain. Here, neither the Δ*rcaC* strain nor the Δ*bolA* strain showed differences in the size or shape of carboxysomes between RL and GL (Fig. 7A; see also Fig. 8A and B). Since the WT exhibited a decrease in carboxysome diameter and trended toward higher carboxysome abundance under GL conditions, both the Δ*rcaC* and Δ*bolA* strains had significantly larger and fewer carboxysomes than were seen in the WT under GL conditions.

Under conditions of increasing light intensity, the WT showed a gradual increase in carboxysome diameter that was significant in comparisons of HL to LL (*P* = 0.024, Fig. 8C; see also Table 1) and no increase in carboxysome abundance (Fig. 8D). The Δ*rcaE* mutant showed a similar increasing trend in carboxysome diameter, with HL-acclimating cultures showing a significant increase in size (*P* < 0.001 [comparing HL to either ML or LL]) (Fig. 8C). Unlike the WT, the Δ*rcaE* mutant exhibited substantial increases in carboxysome numbers when responding to increased light. The Δ*rcaE* mutant did not exhibit its characteristic increase in carboxysome abundance compared to the WT until it was acclimated to ML or HL under WL growth conditions (Fig. 8D).

**TABLE 1:** Quantification of average carboxysome sizes and numbers per cell section

Condition^*a*^	*F.* *diplosiphon* strain	Carboxysome size (nm)^*b*^	No. of carboxysomes/ cell section^*b*^	No. of samples used for carboxysome size measurements	No. of sections used for carboxysome/ cell section measurements
RL	WT	340 ± 19	3.0 ± 0.4	27	30
	Δ*rcaE*	224 ± 12*	6.2 ± 0.6*	43	30
	Δ*rcaC*	323 ± 27	1.9 ± 0.3*	24	30
	Δ*bolA*	345 ± 15	2.5 ± 0.3	28	30

GL	WT	227 ± 19#	3.8 ± 0.3	45	30
	Δ*rcaE*	174 ± 5*,#	7.2 ± 0.9*	106	30
	Δ*rcaC*	325 ± 26*	2.0 ± 0.3*	18	30
	Δ*bolA*	336 ± 18*	2.6 ± 0.3*	31	30

LL	WT	318 ± 26	1.9 ± 0.3	26	30
	Δ*rcaE*	155 ± 9*	1.9 ± 0.4	34	30

ML	WT	354 ± 23	1.8 ± 0.3	21	30
	Δ*rcaE*	166 ± 7*	3.5 ± 0.6*,#	61	30

HL	WT	404 ± 25#	1.6 ± 0.3	19	30
	Δ*rcaE*	236 ± 11*,#	5.2 ± 0.6*,#	66	30

C_i_ upshift	WT	436 ± 19	1.4 ± 0.1	42	60
	Δ*rcaE*	171 ± 7*	3.7 ± 0.4*	95	60

Air	WT	362 ± 15#	2.1 ± 0.2#	66	60
	Δ*rcaE*	244 ± 10*,#	4.5 ± 0.5*	134	60

C_i_ downshift	WT	332 ± 27#	1.3 ± 0.2	22	30
	Δ*rcaE*	211 ± 14*,#	3.4 ± 0.5*	52	30

a The parameters listed indicate the conditions under which F. diplosiphon cells were grown as described in Methods and Materials (i.e., RL, red light at ∼10 to 12 μmol m^−2^ s^−1^; GL, green light at ∼10 to 12 μmol m^−2^ s^−1^; LL, low GL-enriched WL at 12 μmol m^−2^ s^−1^; ML, medium GL-enriched WL at 30 μmol m^−2^ s^−1^; HL, high GL-enriched WL at 100 μmol m^−2^ s^−1^; C_i_ upshift, air enriched with 3% CO_2_; Air, ambient air; C_i_ downshift, growth under air enriched with 3% CO_2_, followed by a shift to ambient air for ∼19 h.

b Numbers for carboxysome size and carboxysomes/cell section are presented as averages ± SE. Comparisons subjected to statistical analyses using a Student's *t* test that resulted in *P* values of <0.05 are indicated as follows: *, mutant versus WT under the same conditions; #, significant difference versus reference condition (RL, LL, or C_i_ upshift) for the same strain.

C_i_ availability also impacted carboxysome morphology as expected. While the WT strain showed a characteristic decrease in carboxysome abundance under conditions of C_i_ upshift (Fig. 8F), it also showed an increase in carboxysome diameter (Fig. 8E) (same data as reported in Lechno-Yossef et al. [54]). The C_i_ downshift conditions did not provide sufficient time for complete carboxysome acclimation, which takes 2 to 4 days for Synechococcus elongatus sp. PCC 7942 (here referred to as S. elongatus) (5). While the WT strain under conditions of C_i_ downshift showed carboxysome abundance levels similar to those seen under C_i_ upshift conditions, it exhibited decreased carboxysome size (*P* = 0.003), which could in part have been due to the transition to the air-acclimated state (Fig. 8E and F). Overall, the Δ*rcaE* mutant showed a misregulated response to C_i_ availability and a decrease in carboxysome diameter in response to C_i_ upshift (compared to the increase seen in the WT; Fig. 8E) and no significant response with respect to carboxysome abundance (Fig. 8F).

### Transcriptional regulation of CCM components measured by quantitative PCR (qPCR) analysis.

Given that multiple components of the CCM are expected to be controlled at the transcriptional level in response to light and C_i_ availability (4, 27, 29, 30, 55) and the observed changes in carboxysome size for the strains described above, we anticipated changes in regulation of *ccm* genes under the tested conditions. Thus, we analyzed the CCM components of the F. diplosiphon transcriptome using quantitative PCR (qPCR) analysis (see Table 2 for gene-specific primers). These analyses included carboxysome-related genes in the *ccmK1K2LMNO* and *ccmK3K4* operons; *ccmK6*, *ccmP*, *rbcL* and *rbcS* (the RubisCO subunits); *ccaA1*/*2* (carboxysomal CA); and *alc* (the homologue of the RubisCO activase gene [54]). Genes related to C_i_ uptake were also probed, including low-C_i_-induced *cmpA* (BCT complex), *sbtA*, and *ndhD3* (NDH-I_3_ complex); constitutively expressed *ndhD4* (NDH-I_4_ complex) and *bicA*; and a LysR-type transcriptional regulator with homology to *cmpR* (56) and *ccmR* (6), the latter two of which are each involved in the transcriptional response to C_i_ availability.

**TABLE 2:** Primers used for qPCR probes

Target gene	Forward primer (5′–3′)	Reverse primer (5′–3′)
*ccmK1*	AACGAATTGGCAGGACATACT	GCAGGCGTAGAATCTGTGAA
*ccmK2*	AGGCTTGCACTTCCGATAC	TGCTGATGCGATGGTGAA
*ccmK3*	TGCTGCTGGAGAACAAGTAAA	GTAAAGTGGATCGGAAGGATGG
*ccmK4*	CAGGCAGTTGGAGCATTAGA	TCAGAAACATCGCCACGAATA
*ccmK6*	GAAGCAGTAGGACGAGTGAATG	ATTGGCGCTGCGATGAA
*ccmL*	GTCTACTCCTGCACCTACGATA	GTCTTCGAGGTGTGAAACTACTG
*ccmM*	GCAACAAGCTGACCGTTTAC	CTATCTGCAACGCACAAATATCC
*ccmN*	TGGCACTCAGATTTATGGTACAG	GTCCGAGATGGGTTCATTTAGAG
*ccmO*	CCATTACCTCCAAGCTCAGTAAA	CTCCTACCATCGCTGGAAATC
*ccmP*	TCATTCTAGCTCTCAAGGAGAAAC	CTAGAAACAACCCGAGGCTTTA
*ccaA1*	GCTCAAGTATACAGAGGCAACC	GAGTCAGTACATTCTCCGCAATAA
*ccaA2*	AACGAGCAGTTCGATTACCC	ATGCGCTCCCATTGTTCT
*alc*	CCGGCAACTATTCCTACCTTATC	TCGTGACAGGCAACGATTT
*rbcL*	GTTAGAAGGTGAGCGTGGTATC	GAAGCCCAGTCTTGGGTAAA
*rbcS*	TGTTCGGCGCTAAATCTACTC	GCTTGATGTTGTCAAAGCCTAC
LysR type	TCGGTCGGATTGCCTTTATTT	GCCGACAAGTAGCAAACAATTC
*cmpA*	CTGCATTAACCGCAGAGATTTG	GAGTATTGCTTTGGTGGCTTTG
*sbtA*	GTGGAACTGCGATCCGTAAT	ATGTATAGCGGGCGATGAATAC
*ndhD3*	TTCTCAGCGTTTCCCATCTC	CAGGTACGGTTGAGAAGAATCA
*ndhD4*	TGACTGCCGTGTACTTCTTAATC	GTAGGCGATCGCTCCAATATAC
*bicA*	GTTGCGGTTTGTACCGAATATG	TGTGGCTGTAAACCTGTGAG
*orf10B*	AGAACTACAGCGTCAGCTTAAT	CTGCTTCGCTTTCAGCATTT

We hypothesized that the photoregulation of CCM components might correspond to the changes in carbon assimilation described above. Thus, we first analyzed strains under RL and GL conditions (Table 3). Whereas the Δ*rcaE* mutant showed upregulation of *ccmM* and downregulation of *rbcS* under RL conditions, more-significant changes were observed under GL conditions, particularly in the downregulation of *ccmK3*, *rbcL*, *rbcS*, and the low-C_i_ induced C_i_-uptake genes relative to the WT. The regulation of *ccmM*, *rbcL*, and *rbcS* was consistent with prior results (29), as was the downregulation of *sbtA* and *ndhD3* (55). The WT showed few differences between RL and GL conditions; however, *alc*, *bicA*, and *cmpA* were downregulated under GL conditions. For many genes, the Δ*rcaE* mutant also exhibited downregulation under GL conditions but with more extreme and more frequently statistically significant magnitudes of change. The Δ*rcaC* mutant showed almost no differences under RL versus GL conditions except a failure to downregulate *alc* under GL conditions. Finally, the Δ*bolA* mutant showed downregulation of *ccmK2*, *ccmK3*, *ccmK4*, and *sbtA* under RL conditions.

**TABLE 3:** Relative expression levels of *ccm* genes under RL versus GL conditions in *F. diplosiphon* strains^*a*^

Gene	Relative expression (−Δ*C_q_* ± SD) in indicated *F. diplosiphon* strain							
	RL				GL			
	WT (*n* = 5)	Δ*rcaE* (*n* = 5)	Δ*rcaC* (*n* = 5)	Δ*bolA* (*n* = 6)	WT (*n* = 5)	Δ*rcaE* (*n* = 5)	Δ*rcaC* (*n* = 6)	Δ*bolA* (*n* = 6)
*ccmK1*	6.1 ± 0.4	6.7 ± 0.8	5.9 ± 0.5	5.7 ± 0.5	5.9 ± 0.4	6.0 ± 0.4	6.3 ± 0.5	6.2 ± 0.1
*ccmK2*	6.1 ± 0.4	6.8 ± 0.8	6.0 ± 0.4	5.5 ± 0.5*	5.8 ± 0.4	6.0 ± 0.2	6.2 ± 0.5	5.9 ± 0.2
*ccmK3*	5.1 ± 0.3	5.6 ± 0.5	5.0 ± 0.4	4.5 ± 0.3*	5.0 ± 0.2	4.4 ± 0.2*,#	5.1 ± 0.6	4.9 ± 0.2#
*ccmK4*	5.3 ± 0.3	5.5 ± 0.6	5.3 ± 0.4	4.7 ± 0.3*	5.0 ± 0.4	4.8 ± 0.4	5.6 ± 0.7	5.1 ± 0.3#
*ccmK6*	−0.5 ± 0.3	−0.3 ± 0.4	−0.4 ± 0.4	−0.1 ± 0.6	−0.7 ± 0.3	−1.0 ± 0.4#	−0.2 ± 0.6	−0.4 ± 0.1
*ccmL*	4.9 ± 0.6	5.5 ± 0.7	4.8 ± 0.4	4.3 ± 0.6	4.6 ± 0.3	4.4 ± 0.3#	4.9 ± 0.4	4.7 ± 0.2
*ccmM*	5.1 ± 0.5	6.3 ± 0.1*	5.2 ± 0.5	4.6 ± 0.6	5.2 ± 0.2	5.0 ± 0.3#	5.4 ± 0.5	5.0 ± 0.2
*ccmN*	3.8 ± 0.8	4.1 ± 0.5	3.4 ± 0.4	3.1 ± 0.6	3.5 ± 0.3	3.2 ± 0.2#	3.7 ± 0.3	3.5 ± 0.2
*ccmO*	3.8 ± 1.3	2.8 ± 0.7	3.5 ± 0.6	3.3 ± 0.8	3.5 ± 0.6	2.8 ± 0.2	3.6 ± 0.4	3.7 ± 0.1
*ccmP*	0.1 ± 0.5	0.5 ± 0.3	0.2 ± 0.2	0.2 ± 0.4	−0.2 ± 0.4	−0.4 ± 0.3#	0.2 ± 0.4	0.2 ± 0.1
*ccaA1*	−0.1 ± 0.7	−0.3 ± 0.7	0.5 ± 0.6	1.2 ± 1.2	−0.1 ± 0.2	−0.1 ± 0.4	0.0 ± 0.2	−0.1 ± 0.3#
*ccaA2*	−1.2 ± 0.7	−1.4 ± 0.6	−0.7 ± 0.3	0.1 ± 1.1*	−1.3 ± 0.4	−1.2 ± 0.5	−1.2 ± 0.3#	−1.0 ± 0.3
*alc*	3.0 ± 0.2	3.0 ± 0.6	2.7 ± 0.4	3.3 ± 0.5	2.2 ± 0.2#	1.7 ± 0.4#	2.6 ± 0.2*	2.9 ± 0.1*
*rbcL*	6.7 ± 0.9	6.0 ± 0.5	6.6 ± 0.7	5.5 ± 0.6*	6.4 ± 0.3	5.3 ± 0.3*,#	6.6 ± 0.5	6.0 ± 0.3
*rbcS*	7.1 ± 0.7	4.5 ± 0.1*	6.4 ± 0.6	5.8 ± 0.4*	6.8 ± 0.1	4.7 ± 0.2*	6.8 ± 0.3	6.2 ± 0.3*
LysR type	1.6 ± 0.5	1.3 ± 0.5	1.6 ± 0.4	1.4 ± 0.3	1.3 ± 0.4	1.5 ± 0.3	1.7 ± 0.3	1.6 ± 0.1
*cmpA*	1.8 ± 1.5	0.3 ± 0.5	0.0 ± 0.9	0.5 ± 0.6	−1.4 ± 0.4#	−2.3 ± 0.5*,#	−1.0 ± 0.6	−1.2 ± 0.3#
*sbtA*	3.9 ± 0.7	4.3 ± 0.5	4.1 ± 1.1	2.8 ± 0.6*	4.5 ± 0.7	2.5 ± 0.4*,#	4.3 ± 0.6	4.0 ± 0.5#
*ndhD3*	3.9 ± 0.5	3.9 ± 0.8	4.0 ± 0.6	3.5 ± 0.2	4.1 ± 0.2	2.7 ± 0.4*,#	4.3 ± 0.4	4.3 ± 0.4#
*ndhD4*	2.7 ± 0.3	3.2 ± 0.6	2.5 ± 0.4	2.2 ± 0.5	2.6 ± 0.2	2.6 ± 0.4	2.6 ± 0.3	2.6 ± 0.3
*bicA*	0.4 ± 0.3	0.5 ± 0.6	0.4 ± 0.4	0.8 ± 0.6	0.0 ± 0.2#	0.1 ± 0.3	0.3 ± 0.3	0.3 ± 0.3

a qPCR expression data represent WT, Δ*rcaE*, Δ*rcaC*, and Δ*bolA* F. diplosiphon strains grown under RL or GL (∼10 to 12 μmol·m^−2^·s^−1^) conditions. Data for each gene are presented as −Δ*C_q_* ± standard deviation (SD) relative to the endogenous control gene *orf10B* and represent a log_2_ scale. Comparisons subjected to statistical analyses using a Student's *t* test that resulted in *P* values of <0.05 are indicated as follows: *, mutant versus WT under the same condition; #, GL versus RL in the same strain.

Under conditions of increasing light intensity (Table 4), WT experienced significant upregulation for selected HCO_3_- transporter genes (likely due to increased linear electron flow), *ccmN*, and *ccmO*, alongside downregulation for *rbcS* (possibly related to HL stress). The Δ*rcaE* mutant showed the characteristic downregulation of *rbcS* that was seen under other conditions. Additionally, it exhibited upregulation of *ccmK1* and *ccmK2* under ML conditions and of *ccmK6* under HL conditions, which correlates with the increase in carboxysome abundance (Fig. 7B; see also Fig. 8D). The Δ*rcaE* mutant showed a similar upregulation of HCO_3_- transporter genes, *ccmN*, and *ccmO*, though not to the same extent as the WT. Finally, in contrast to the nonsignificant increases seen in WT, the Δ*rcaE* mutant showed significant upregulation of *alc*. Since the *alc* gene is important for cellular responses to C_i_ upshift (54), this upregulation might be indicative of altered C_i_ utilization by Δ*rcaE* cells.

**TABLE 4:** Relative expression levels of *ccm* genes under conditions of increasing light intensity^*a*^

Gene	Relative expression (−Δ*C_q_* ± SD) in indicated *F. diplosiphon* strain					
	LL		ML		HL	
	WT (*n* = 4)	Δ*rcaE* (*n* = 4)	WT (*n* = 3)	Δ*rcaE* (*n* = 3)	WT (*n* = 5)	Δ*rcaE* (*n* = 5)
*ccmK1*	6.7 ± 0.4	6.9 ± 0.8	7.1 ± 0.3	8.2 ± 0.3*,#	6.1 ± 0.8	7.1 ± 0.9
*ccmK2*	6.6 ± 0.6	6.9 ± 0.8	6.8 ± 0.2	7.7 ± 0.4*	5.9 ± 0.8	6.9 ± 0.9
*ccmK3*	5.6 ± 0.7	5.2 ± 0.7	5.7 ± 0.4	5.7 ± 0.4	4.7 ± 0.5	5.2 ± 0.5
*ccmK4*	5.5 ± 0.2	5.3 ± 0.5	5.8 ± 0.2	5.9 ± 0.4	5.3 ± 0.5	5.7 ± 0.7
*ccmK6*	−0.5 ± 0.2	−0.8 ± 0.4	−0.5 ± 0.2	−0.4 ± 0.2	−0.4 ± 0.4	0.1 ± 0.3#
*ccmL*	5.4 ± 0.5	5.3 ± 0.7	6.0 ± 0.5	6.6 ± 0.1#	4.8 ± 0.7	5.7 ± 0.8
*ccmM*	5.7 ± 0.4	5.7 ± 0.8	6.4 ± 1.1	7.2 ± 0.2#	5.1 ± 0.5	6.2 ± 0.5*
*ccmN*	3.7 ± 0.2	3.9 ± 0.7	3.9 ± 0.3	4.7 ± 0.4	6.9 ± 1.1#	5.3 ± 0.8*,#
*ccmO*	3.5 ± 0.2	3.3 ± 0.3	3.9 ± 0.1#	3.7 ± 0.2	7.8 ± 1.1#	6.4 ± 1.1#
*ccmP*	0.4 ± 0.2	0.3 ± 0.3	0.6 ± 0.1	0.7 ± 0.2#	0.1 ± 0.5	0.5 ± 0.4
*ccaA1*	0.3 ± 0.6	0.0 ± 0.7	0.0 ± 0.5	−0.3 ± 0.7	0.3 ± 1.2	0.1 ± 0.5
*ccaA2*	−1.0 ± 0.5	−1.4 ± 0.4	−1.0 ± 0.1	−1.2 ± 0.5	−0.4 ± 1.5	−0.6 ± 0.8
*alc*	2.6 ± 0.5	2.2 ± 0.7	3.5 ± 0.4	3.5 ± 0.3#	2.8 ± 0.8	3.3 ± 0.5#
*rbcL*	7.6 ± 0.7	6.4 ± 1.0	8.5 ± 1.7	8.0 ± 1.2	7.1 ± 1.1	7.0 ± 1.0
*rbcS*	7.9 ± 0.2	5.0 ± 0.3*	8.0 ± 0.9	5.4 ± 0.1*	6.6 ± 0.4#	4.5 ± 1.4*
LysR−type	2.0 ± 0.4	1.8 ± 0.6	2.4 ± 0.4	2.5 ± 0.4	4.1 ± 0.2#	3.1 ± 0.4*,#
*cmpA*	−1.6 ± 0.5	−1.8 ± 0.8	−0.6 ± 0.3#	−0.4 ± 0.5#	5.8 ± 0.3#	4.3 ± 1.1*,#
*sbtA*	4.5 ± 0.2	3.5 ± 0.8	5.1 ± 0.6	4.3 ± 0.5	6.2 ± 0.2#	5.9 ± 0.7#
*ndhD3*	4.0 ± 0.6	3.3 ± 1.0	5.0 ± 0.5	4.6 ± 0.3	4.5 ± 0.3	5.0 ± 1.1
*ndhD4*	2.6 ± 0.6	2.9 ± 0.8	3.3 ± 0.3	3.7 ± 0.4	2.7 ± 0.2	3.2 ± 0.8
*bicA*	−0.6 ± 0.8	−0.2 ± 0.7	0.5 ± 0.2	0.3 ± 0.7	0.7 ± 0.4#	1.6 ± 0.6*,#

a qPCR expression data represent WT and Δ*rcaE F. diplosiphon* strains grown under conditions of LL (12 μmol·m^−2^·s^−1^), ML (30 μmol·m^−2^·s^−1^), or HL (100 μmol·m^−2^·s^−1^) GL-enriched WL intensity. Data for each gene are presented as −Δ*C_q_* ± standard deviation (SD) relative to the endogenous control gene *orf10B* and represent a log_2_ scale. Comparisons subjected to statistical analyses using a Student's *t* test that resulted in *P* values of <0.05 are indicated as follows: *, Δ*rcaE* strain versus WT under the same condition; #, significant difference versus LL in the same strain.

Both the WT and Δ*rcaE* strains demonstrated significant differential expression of CCM components under conditions of decreasing C_i_ availability (Table 5). The WT showed a general downregulation in shell protein genes, *rbcL*, *rbcS*, and *ccmM* under conditions of C_i_ downshift, which is consistent with previous findings for *Synechocystis* (6, 57) and S. elongatus (58). It is interesting to consider how these data correlate with the increased carboxysome abundance under conditions of C_i_ downshift reported previously (5, 57, 59) and in this study (Fig. 8E and F; C_i_ upshift versus air). As previously noted (54), *alc* is downregulated under conditions of C_i_ downshift and has been observed to be involved in decreased carboxysome abundance under conditions of C_i_ upshift. Consistent with these expectations, WT also exhibited significant upregulation of the low-C_i_-induced C_i_-uptake genes.

**TABLE 5:** Relative expression levels of *ccm* genes under conditions of decreasing carbon availability^*a*^

Gene	Relative expression (−Δ*C_q_* ± SD) in indicated *F. diplosiphon* strain					
	C_i_ upshift		Air		C_i_ downshift	
	WT (*n* = 5)	Δ*rcaE* (*n* = 4)	WT (*n* = 5)	Δ*rcaE* (*n* = 4)	WT (*n* = 5)	Δ*rcaE* (*n* = 4)
*ccmK1*	6.9 ± 0.6	7.7 ± 0.4	6.1 ± 0.3#	6.7 ± 0.6#	6.2 ± 0.3	6.8 ± 0.5#
*ccmK2*	7.0 ± 0.5	7.6 ± 0.3	6.0 ± 0.3#	6.5 ± 0.5#	5.8 ± 0.3#	6.4 ± 0.5#
*ccmK3*	5.4 ± 0.3	5.2 ± 0.5	4.2 ± 0.3#	4.9 ± 0.1*	4.1 ± 0.2#	4.2 ± 0.5#
*ccmK4*	5.8 ± 0.3	5.6 ± 0.5	4.8 ± 0.2#	5.3 ± 0.2*	4.7 ± 0.2#	4.6 ± 0.4#
*ccmK6*	0.3 ± 0.3	−0.1 ± 0.3	−0.4 ± 0.4#	0.0 ± 0.3	−0.7 ± 0.5#	−0.4 ± 0.4
*ccmL*	5.7 ± 0.3	6.4 ± 0.4*	4.9 ± 0.2#	5.4 ± 0.5#	4.8 ± 0.2#	5.3 ± 0.4#
*ccmM*	6.5 ± 0.2	7.1 ± 0.5	5.1 ± 0.2#	5.9 ± 0.7#	4.7 ± 0.1#	5.3 ± 0.6#
*ccmN*	3.7 ± 0.3	4.8 ± 0.2*	5.4 ± 0.5#	3.8 ± 0.8*	7.4 ± 0.5#	7.2 ± 0.8#
*ccmO*	2.8 ± 0.4	3.9 ± 0.3*	6.2 ± 0.5#	2.7 ± 0.8*,#	8.0 ± 0.4#	7.8 ± 0.8#
*ccmP*	0.8 ± 0.3	1.0 ± 0.5	−0.1 ± 0.5#	0.6 ± 0.3*	−0.9 ± 0.3#	−0.7 ± 0.7#
*ccaA1*	−1.1 ± 0.2	−0.8 ± 0.2	−0.5 ± 0.4#	−0.3 ± 1.1	−0.9 ± 0.5	−0.4 ± 0.5
*ccaA2*	−1.9 ± 0.4	−1.8 ± 0.2	−1.4 ± 0.5	−1.3 ± 0.9	−2.2 ± 0.4	−1.7 ± 0.7
*alc*	3.8 ± 0.5	3.2 ± 0.6	2.5 ± 0.2#	3.3 ± 0.8	1.7 ± 0.3#	2.1 ± 0.6#
*rbcL*	8.3 ± 0.3	7.4 ± 0.3*	7.1 ± 0.6#	5.6 ± 0.8*,#	7.5 ± 0.4#	7.5 ± 0.5
*rbcS*	8.3 ± 0.5	5.6 ± 0.5*	7.5 ± 0.5#	4.2 ± 0.4*,#	7.6 ± 0.4	6.7 ± 0.3*,#
LysR−type	2.1 ± 0.5	2.7 ± 0.2*	3.4 ± 0.2#	1.9 ± 0.7*	3.8 ± 0.3#	3.4 ± 0.2#
*cmpA*	−4.3 ± 0.4	−3.3 ± 0.4*	6.2 ± 0.2#	−1.9 ± 0.3*,#	6.6 ± 0.5#	6.7 ± 0.3#
*sbtA*	0.2 ± 0.3	0.2 ± 0.6	4.5 ± 0.3#	1.8 ± 0.2*,#	5.1 ± 0.6#	5.3 ± 0.3#
*ndhD3*	3.8 ± 0.3	3.8 ± 0.6	4.7 ± 0.3#	3.1 ± 1.1	5.4 ± 0.5#	6.1 ± 0.4*,#
*ndhD4*	3.0 ± 0.2	3.8 ± 0.3*	2.3 ± 0.2#	2.9 ± 0.8	2.6 ± 0.3#	2.9 ± 0.6
*bicA*	0.7 ± 0.2	0.7 ± 0.3	0.8 ± 0.3	1.4 ± 0.2*,#	0.5 ± 0.2	1.3 ± 0.3*,#

a qPCR expression data represent WT and Δ*rcaE* F. diplosiphon strains grown under conditions of C_i_ upshift (3% CO_2_), air, or C_i_ downshift (19 h after a transfer from 3% CO_2_ to air). Data for each gene are presented as −Δ*C_q_* ± standard deviation (SD) relative to the endogenous control gene *orf10B* and represent a log_2_ scale. Comparisons subjected to statistical analyses using a Student's *t* test that resulted in *P* values of <0.05 are indicated as follows: *, Δ*rcaE* strain versus WT under the same condition; #, significant difference versus C_i_ upshift in the same strain.

While the WT upregulated the low-C_i_-induced C_i_-uptake genes under both air and C_i_-downshift conditions, the Δ*rcaE* mutant did so only under conditions of C_i_ downshift. Nevertheless, *ccm* gene transcription in the Δ*rcaE* strain was similar to that seen with the WT under conditions of both C_i_ upshift and downshift overall, with the major differences occurring when the two strains transitioned from one carbon status to the other. Notably, the Δ*rcaE* mutant also recovered near-WT levels of *rbcS* under conditions of C_i_ downshift, possibly explaining the strain’s recovery of assimilation under those conditions.

## DISCUSSION

### Use of the CRC in cyanobacteria.

Our work with F. diplosiphon, a freshwater filamentous cyanobacterium which undergoes CCA in response to light quality, highlights multilayered connections between CCM components, nutrient availability, and the physiological state of the cell (29). Efficiently connecting these factors to overall carbon assimilation is critical to understanding how these organisms (and humans as bio-prospectors) can optimize photosynthesis. We hypothesized that identification of the conditions under which carbon assimilation was disrupted in WT F. diplosiphon or a Δ*rcaE* mutant strain with compromised CCA would highlight functional roles of CCA in impacting the regulation of CCM and associated carbon fixation and would indicate mechanisms for future analysis.

The use of gas exchange analysis to construct CRCs in cyanobacteria suggests that the acclimation to dominant light quality through CCA has a nuanced impact on overall assimilation behavior. WT F. diplosiphon cells assimilate more CO_2_ when acclimated to GL despite having smaller carboxysomes and not being tuned to the red-enriched light of the Li-COR system (Fig. 2C). The disruption of CCA through the loss of the photoreceptor RcaE added layers of complexity; since RcaE influences the stoichiometry of carboxysome components and carboxysome size under both RL and GL conditions (29), we expected a general decrease in net CO_2_ uptake and assimilation. Instead, we found GL-specific impairment (Fig. 2D). While the small, more numerous carboxysomes of the Δ*rcaE* strain may contribute to overall carbon assimilation behavior, this observation cannot explain the higher level of assimilation seen with the WT under GL conditions. These intriguing initial results prompted further exploration of the assimilation behavior of cyanobacteria.

We provide evidence that physiologically relevant CRCs, similarly to the popular carbon assimilation-versus-intracellular CO_2_ curves in plants, can be obtained from cyanobacteria in a semiwet state using cyanobacterial discs. Cells showed a dosage response to both light (Fig. 2A) and CO_2_, two major factors that are relevant to the development of the advanced modeling of photosynthetic parameters in plants (41). CRCs were also sensitive enough to show changes in apparent compensation points based on the physiological state of the cell (Fig. 4C). Traditional O_2_ evolution experiments revealed similar trends, with the WT exhibiting higher rates under GL than RL conditions and the Δ*rcaE* mutant showing higher rates under RL than GL conditions (Fig. 6). Despite this, the two methods differed in comparisons of the WT and Δ*rcaE* strains under RL conditions; the Δ*rcaE* mutant exhibited similar C_i_-uptake rates under RL conditions but a decrease in O_2_ evolution, suggesting an impairment in the use of CO_2_ for oxygenic photosynthesis in the Δ*rcaE* mutant. Thus, CRCs of cyanobacterial discs offer novel insight into the CO_2_-uptake behavior of cyanobacteria under a broad range of C_i_ levels. This method also significantly reduces the time required for equilibration between CO_2_ and HCO_3_-, which allows dynamic responses to be studied. Thus, it is a promising technique that can be used both as a stand-alone method as a quick measurement of net carbon assimilation and in conjunction with established systems that more deeply probe HCO_3_-/CO_2_ flux. In particular, and in contrast to well-established procedures that test cyanobacteria’s utilization of HCO_3_-, it serves to more directly test the use of CO_2_ by cyanobacteria.

### The low-C_i_ phase of the CRC (≤100 ppm s[CO_2_]) is driven by C_i_ uptake.

The idea of the presence of a C_i_-limited region at low ppm s[CO_2_] is supported by data corresponding to the regions of CRCs that do not respond to nonsaturating light at 0 to ∼100 ppm s[CO_2_] (Fig. 3A to D) and is consistent with findings reported previously by Douchi et al. (33). Notably, the low-C_i_ region is considerably robust and rarely exhibits differences; e.g., the Δ*rcaE* mutant is always indistinguishable from the WT in this region.

There were only two conditions under which we observed changes to the low-C_i_ region. The slope and compensation point were incredibly responsive to acclimation of the culture to different C_i_ availabilities, with growth under C_i_ downshift conditions prompting a robust assimilation response even at very low C_i_ levels and a reduced apparent compensation point (Fig. 4C and D). We were tempted to identify this as a light-independent region and so tested a hypothesis predicting that cultures acclimated to C_i_ downshift would not show a change in slope below ∼100 ppm s[CO_2_], even analyzed under nonsaturating light conditions. However, nonsaturating light reduced the assimilation slope and increased the compensation point (Fig. 5C and D). This observation suggests that light availability can affect the low-C_i_ region but only under specific conditions that are related to C_i_-uptake capacity. Thus, we propose identifying the low-C_i_ region of the cyanobacterial CRC as one that is driven by C_i_ uptake and that is comparable to C_i_-limited regions of response curves in plants.

### The high-C_i_ phase of the CRC (≥100 ppm s[CO_2_]) is responsive to multiple photosynthetic parameters.

In line with biphasic models of carbon assimilation in C_4_ plants (42, 43) and cyanobacteria (33), our work supports the identification of a second region that reaches *A*_max_ at high C_i_. However, these data suggest that the high-C_i_ region of cyanobacteria CRCs depends on many variables, including C_i_ availability, carboxysome morphology, linear electron flow, and cell shape.

**FIG 9:**
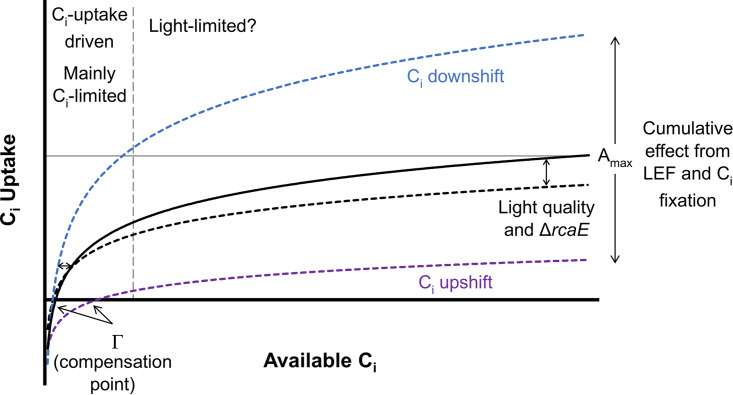
Generalized diagram for proposed interpretation of carbon response curve (CRC) behavior of carbon assimilation in F. diplosiphon. General responses of carbon assimilation to C_i_ availability under a variety of conditions. The solid black curve represents a sigmoidal function that describes standard CRC behavior with a saturation point at *A*_max_ (horizontal gray line). Values of *A*_max_ have been shown to depend on light saturation during the CRC run, linear electron flow (LEF), and acclimation to changes in light intensity and C_i_ availability. The vertical dashed gray line represents an approximate boundary of the biphasic model, with acclimation to C_i_ availability being the primary factor impacting the region left of the boundary (C_i_ uptake driven). The effects of acclimation to C_i_ upshift or downshift are represented in purple and blue dashed lines, respectively. The compensation point, Γ, where the rates of photosynthetic CO_2_ flux and respiration are equivalent, would be the *x* intercept point where the *y* axis is in A (μmol CO_2_ m^−2^ s^−1^) and the *x* axis is the intracellular [CO_2_] around RubisCO. Axis data represent generalized units, as many measurements (both aqueous and gaseous) follow the same trends in cyanobacteria but are difficult to interconvert.

The components of the CCM that relate to C_i_ uptake appear to have a broad effect on assimilation behavior, consistent with the C_i_ upshift results reported by Douchi et al. (33). Indeed, upregulation of the low-C_i_-induced genes (Table 5) was correlated with an increase in assimilation at all s[CO_2_] levels (Fig. 4A). Since this increase occurred under C_i_ downshift conditions, where WT carboxysomes had not had sufficient time to acclimate to air conditions (Fig. 8E and F), this is one case where we can neatly attribute a change in assimilation behavior directly to a single major component of CCM (Fig. 9). However, under HL conditions, we saw similar induction of the low-C_i_-induced genes (Table 4) without the corresponding increase in assimilation (Fig. 3E).

Analysis of the Δ*rcaE* mutant strain provides some additional lines of inquiry that may offer insight. Unlike the WT results, elevated light intensity increased the maximum assimilation rates of the mutant (Fig. 3F). This may have been because the mutant experienced a greater overall increase in carboxysome volume in response to HL (Fig. 8C and D), perhaps evidencing the role of the carboxysomes in carbon assimilation behavior as part of a C_i_ fixation parameter. Since the mutant strain maintained a water splitting capacity similar to that seen with WT (Fig. 6; +DCBQ) but showed a decreased net O_2_ evolution rate (Fig. 6; −DCBQ) under RL and GL conditions and decreased *A*_max_ under GL conditions, the Δ*rcaE* mutant was also less efficient at utilizing light productively. Thus, HL conditions would prove beneficial to the mutant (as evidenced by its increase in assimilation) while being stressful to the more efficient WT. This suggests that carboxysome size or linear electron flow or both contribute to the determination of *A*_max_ and are the primary contributors to the low *A*_max_ of the Δ*rcaE* mutant strain (Fig. 9). Second, the behavior of the Δ*rcaE* mutant yields insight into the assimilation phenotype of the WT under GL conditions. Though *cmpA* was downregulated under GL conditions in WL, the Δ*rcaE* mutant showed much more significant downregulation of low-C_i_-induced genes (Table 3), which may contribute to the low-assimilation phenotype, and perhaps to the C_i_-uptake capacity, of the Δ*rcaE* mutant under GL conditions. If this is the case, then it is probable that the inducible C_i_-uptake systems contributed but were being masked in the high-carbon-assimilation phenotype of the WT under GL conditions.

Both the Δ*rcaC* and Δ*bolA* mutants showed few differences between RL and GL in the experiments performed in this study. Under both RL and GL conditions, the Δ*rcaC* strain, which was constitutively in a GL-like phenotypic state, showed nearly identical assimilation behaviors that were more similar to those of the WT under GL conditions (Fig. 2E), suggesting that GL acclimation also contributes to the high-assimilation phenotype of the WT. As for the Δ*bolA* strain, it too showed nearly identical assimilation behavior in both RL and GL but was instead more similar to the WT under RL conditions (Fig. 2F). As the Δ*bolA* mutant had an enlarged, spherical cell shape under both RL and GL conditions, it is possible that the rod shape of WT F. diplosiphon cells seen under GL conditions enhanced C_i_ uptake and/or cellular CO_2_ diffusion.

### Impact.

This study integrated physiological analyses of the cyanobacterium F. diplosiphon with a novel application of gas exchange analysis to cyanobacteria. Like many cyanobacteria, F. diplosiphon performs CCA, which offers a useful system for studying the impact of light regulation, especially as it relates to photosynthesis. We explored the connection between the loss of RcaE, a cyanobacteriochrome that controls the CCA pathway, and the CCM. Analyses of the CRCs provide a simple method to assay the carbon assimilation phenotype of cyanobacteria, connecting findings on how the stoichiometry of CCM components influences the structure and function of carboxysomes and C_i_-uptake systems. Preliminary work to identify photosynthetic parameters that are identifiable through CRCs could contribute valuable insight into modeling and understanding the dynamic regulation of photosynthesis in cyanobacteria.

## MATERIALS AND METHODS

### Growth conditions.

General culture inoculation and growth under RL and GL conditions were performed as described previously by Rohnke et al. (29). In brief, we used a short-filament strain of F. diplosiphon with WT pigmentation identified as SF33 (60), a RcaE-deficient mutant strain (the Δ*rcaE* mutant) characterized previously by Kehoe and Grossman (47), a RcaC-deficient mutant strain (the Δ*rcaC* mutant) identified in our lab through forward genetics screening, and a BolA-deficient mutant strain (the Δ*bolA* mutant) described previously by Singh and Montgomery (48). Liquid cultures were inoculated from plated cultures and grown at 28°C under WL in BG-11/HEPES until they were diluted to an initial OD_750_ of 0.05 and transferred to experimental conditions.

The effect of light intensity was tested in a MultiCultivator MC 1000-OD system (Photon Systems Instruments, Drasov, Czech Republic) equipped with LED WL and autonomous monitoring of OD_680_ and OD_720_ according to the manufacturer’s directions. Since the LED WL was GL dominant, starter cultures grown under GL were used for experiments involving the multicultivator to avoid the WT showing a growth lag as it underwent CCA. Light conditions were set at a constant value of 12 μmol·m^−2^·s^−1^ (LL), 30 μmol·m^−2^·s^−1^ (ML), or 100 μmol·m^−2^·s^−1^ (HL). Since sustained HL conditions ultimately caused chlorosis, when high ODs were needed for harvesting for transmission electron microscopy (TEM) and RNA extraction, the ML and HL cultures were first grown at 12 μmol·m^−2^·s^−1^ for 1 to 2 days prior to the onset of ML and HL conditions. Cultures grown this way were allowed to acclimate to the higher light intensity for at least 3 days prior to harvesting. Cultures from all experiments involving HL-grown cultures were harvested prior to the plateauing of OD (within 6 days of HL onset) that preceded substantial cell death.

The effect of carbon availability was tested in Multitron growth chambers (Infors HT, Bottmingen-Basel, Switzerland) at 30°C under WL (∼35 to 40 μmol·m^−2^·s^−1^, with RL enrichment) gassed with either unenriched air (air) or air enriched with 3% CO_2_ (C_i_ upshift). As described previously by Lechno-Yossef et al. (54) and on the basis of methods described previously by Wang et al. (6), we shifted cultures from C_i_ upshift to air conditions after 3 days of growth and resuspended them in BG11/HEPES that lacked sodium bicarbonate to achieve C_i_ downshift. Cells were harvested for CRC, TEM, or qPCR analysis ∼19 h after transfer to air (C_i_ downshift).

### Carbon response curve analysis using F. diplosiphon discs.

**FIG 10:**
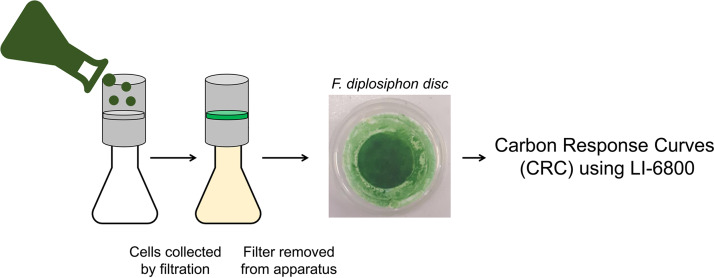
Methodology of the filtered-disc method for CRC analysis of cyanobacteria.

OD_750_ levels were measured in triplicate for cultures growing under the desired experimental conditions and were harvested between the ODs of 0.6 and 1.2. A total volume equal to 11.8 absorbance units (*V* = 11.8/OD_750_) was vacuum filtered through glass fiber filters (Fig. 10) with a pore size that was sufficiently small to capture >99% of F. diplosiphon cells (Whatman GF/A; Sigma-Aldrich, St. Louis, MO) (47-cm diameter) and a second layer of Whatman grade 1 filter paper to diffuse the filtrate more evenly. The disc diameter was selected to minimize unnecessary surplus surface area for the gas exchange chamber; about 47% of the disc’s surface area was exposed to the 6-cm^3^ chamber and barely extended past the gaskets. Cyanobacterial discs were handled carefully with forceps, briefly dabbed on filter paper to remove excess wetness, kept on BG11/HEPES agar plates, and analyzed swiftly to minimize environmental perturbation. CO_2_ levels were measured with infrared gas analysis by the use of Li-COR Photosynthesis System 6800 (Li-COR, Lincoln, NE), with one end of a strip of damp Whatman grade 1 filter paper placed underneath the disc as a wick. The other end was submerged in double-distilled water (ddH_2_O) to maintain disc dampness for the duration of the experiment, which was found to greatly increase the duration during which the steady state could be maintained to ∼45 min (data not shown).

The chamber was illuminated by the use of the standard “Sun+Sky” (RL-dominant) regime with a leaf temperature of 28°C, a flow rate of 500 μmol s^−1^, and a source air with 12 ppm H_2_O. For the standard CRC, the initially supplied CO_2_ concentration was 1,000 ppm and the sample was allowed to equilibrate for at least 5 min or until the steady state had been maintained for at least 3 min. The CRC followed a gradient of 1,000, 850, 700, 550, 400, 300, 200, 150, 100, 75, 50, 25, and 5 ppm, followed by a return to 400 ppm with automatic infrared gas analysis-based matching. The sample was allowed to equilibrate for ∼2 to 3 min at each time point for a total run time of ∼25 min after initial equilibration. Values for *A* were calculated as the loss of CO_2_, in μmol per m^2^ per second, and were corrected for leaks and changes in humidity.

### O_2_ evolution analysis.

O_2_ evolution was measured using an Oxytrace+ O_2_ electrode (Hansatech Instruments Ltd., Norfolk, England) illuminated by an acrylic projector bulb. Illumination was maintained at ∼250 μmol·m^−2^·s^−1^ and measured with a LI-250 light meter (Li-COR) equipped with a quantum sensor (model US-SQS/L; Heinz Walz CmbH, Effeltrich, Germany). Cells containing ∼10 μg Chl*a* (determined on the basis of OD_750_ extinction coefficients [see Fig. S2 in the supplemental material]) were harvested, washed twice in 3 ml BG11/HEPES that lacked sodium bicarbonate, and resuspended in 1 ml BG11/HEPES that lacked sodium bicarbonate. Cyanobacteria were placed in the chamber and spiked with sodium bicarbonate (Sigma-Aldrich) to reach a final concentration of 2 mM prior to illumination. When applicable, 2,6-DCBQ (Sigma-Aldrich) was then added to reach a final concentration of 0.2 mM and with potassium ferricyanide to reach a final concentration of 1.5 mM to act as the terminal electron acceptor. Cells were allowed to equilibrate at ambient light for ∼1.5 min and then illuminated. The O_2_ evolution *V*_max_ was recorded as the peak rate that was reached within 10 min of the commencement of illumination.

### TEM analysis.

For all experimental conditions, TEM analysis was performed according to the methods described previously by Rohnke et al. (29). For the C_i_-upshift and air conditions, 60 cell sections were randomly selected and analyzed for carboxysome numbers in the WT and the Δ*rcaE* mutant, with carboxysome diameters measured in 20 of these sections. In all other strains and under all other conditions, 30 cell sections were analyzed, 10 of which were analyzed for carboxysome diameter, as well. Samples were prepared from at least two independent biological replicates. As a modification to the original method, some samples were analyzed using a JEM 1400 Flash TEM (JEOL USA Inc., Peabody, MA) at an operating voltage of 100 V.

### qPCR analysis.

The abundances of *ccmK1*, *ccmK2*, *ccmK3*, *ccmK4*, *ccmK6*, *ccmL*, *ccmM*, *ccmN*, *ccmO*, *ccmP*, *ccaA1*, *ccaA2*, *alc*, *rbcL*, *rbcS*, fdiDRAFT81170 (a LysR-type transcriptional regulator gene), *cmpA*, *sbtA*, *ndhD3*, *ndhD4*, and *bicA* transcripts were measured relative to the internal control *orf10B* within total RNA extracts from F. diplosiphon strains grown under various experimental conditions and according to previously described research (29, 54) and MIQE guidelines (61). In brief, this involved harvesting ∼20 ml of exponentially growing cells upon reaching the target OD_750_ (∼0.5 to 0.6), handling the samples on ice and flash freezing the cell pellet within 1 h of harvesting, and extracting them with a TRIzol reagent incubated at 95°C, followed by wash steps, DNase treatment (TURBO DNA-free kit; Invitrogen, Madison, WI), and RNA quantification using a NanoDrop ND-1000 Spectrophotometer. Reverse transcription was performed using a qScript cDNA SuperMix kit (Quantabio, Beverly, MA), and qPCR was performed using Fast SYBR green master mix (Applied Biosystems, Foster City, CA) in 384-well plates (Applied Biosystems) with a 10-μl reaction volume, with each procedure performed according to the instructions of the manufacturer. Probe sequences are provided in Table 2. RNA quality was assayed using gel electrophoresis, and genomic contamination was controlled for by verifying that no template-control samples had quantification cycle (*C_q_*) values greater than 5 cycles higher than the respective unknowns. The data reflect three technical replicates for each of at least three independent biological replicates and are presented using the delta *C_q_* method (Δ*C_q_*) in order to foster analyses of comparisons between several strains and conditions.

### Chlorophyll extraction.

Chl*a* was measured spectrophotometrically according to the methods described previously by de Marsac and Houmard (62) for use with F. diplosiphon (63). Samples were harvested in parallel with CRC analysis as a secondary validation of normalization by OD_750,_ and at least three independent biological replicates were analyzed.

### Statistical analysis.

Experiments were performed with *n* ≥ 3 from at least 2 biological replicates for all experiments. Statistical significance was evaluated using Student’s *t* tests performed in R.

## References

[1] Burnap RL, Hagemann M, Kaplan A 2015 Regulation of CO_2_ concentrating mechanism in cyanobacteria. Life (Basel) 5:348–371. doi:10.3390/life5010348. 25636131 PMC4390856

[2] Turmo A, Gonzalez-Esquer CR, Kerfeld CA 2 10 2017, posting date Carboxysomes: metabolic modules for CO_2_ fixation. FEMS Microbiol Lett doi:10.1093/femsle/fnx176. 28934381

[3] Parry MAJ, Keys AJ, Madgwick PJ, Carmo-Silva AE, Andralojc PJ 2008 Rubisco regulation: a role for inhibitors. J Exp Bot 59:1569–1580. doi:10.1093/jxb/ern084. 18436543

[4] Burnap RL, Nambudiri R, Holland S 2013 Regulation of the carbon-concentrating mechanism in the cyanobacterium *Synechocystis* sp. PCC6803 in response to changing light intensity and inorganic carbon availability. Photosynth Res 118:115–124. doi:10.1007/s11120-013-9912-4. 23990371

[5] Sun Y, Casella S, Fang Y, Huang F, Faulkner M, Barrett S, Liu L-N 2016 Light modulates the biosynthesis and organization of cyanobacterial carbon fixation machinery through photosynthetic electron flow. Plant Physiol 171:530–541. doi:10.1104/pp.16.00107. 26956667 PMC4854705

[6] Wang H-L, Postier BL, Burnap RL 2004 Alterations in global patterns of gene expression in *Synechocystis* sp. PCC 6803 in response to inorganic carbon limitation and the inactivation of ndhR, a LysR family regulator. J Biol Chem 279:5739–5751. doi:10.1074/jbc.M311336200. 14612435

[7] Price GD, Woodger FJ, Badger MR, Howitt SM, Tucker L 2004 Identification of a SulP-type bicarbonate transporter in marine cyanobacteria. Proc Natl Acad Sci U S A 101:18228–18233. doi:10.1073/pnas.0405211101. 15596724 PMC539743

[8] Shibata M, Ohkawa H, Kaneko T, Fukuzawa H, Tabata S, Kaplan A, Ogawa T 2001 Distinct constitutive and low-CO_2_-induced CO_2_ uptake systems in cyanobacteria: genes involved and their phylogenetic relationship with homologous genes in other organisms. Proc Natl Acad Sci U S A 98:11789–11794. doi:10.1073/pnas.191258298. 11562454 PMC58809

[9] Zhang P, Battchikova N, Jansen T, Appel J, Ogawa T, Aro E-M 2004 Expression and functional roles of the two distinct NDH-1 complexes and the carbon acquisition complex NdhD3/NdhF3/CupA/Sll1735 in *Synechocystis* sp PCC 6803. Plant Cell 16:3326–3340. doi:10.1105/tpc.104.026526. 15548742 PMC535876

[10] Shibata M, Katoh H, Sonoda M, Ohkawa H, Shimoyama M, Fukuzawa H, Kaplan A, Ogawa T 2002 Genes essential to sodium-dependent bicarbonate transport in cyanobacteria: function and phylogenetic analysis. J Biol Chem 277:18658–18664. doi:10.1074/jbc.M112468200. 11904298

[11] Omata T, Price GD, Badger MR, Okamura M, Gohta S, Ogawa T 1999 Identification of an ATP-binding cassette transporter involved in bicarbonate uptake in the cyanobacterium *Synechococcus* sp. strain PCC 7942. Proc Natl Acad Sci U S A 96:13571–13576. doi:10.1073/pnas.96.23.13571. 10557362 PMC23989

[12] Maeda S, Badger MR, Price GD 2002 Novel gene products associated with NdhD3/D4-containing NDH-1 complexes are involved in photosynthetic CO_2_ hydration in the cyanobacterium, *Synechococcus* sp. PCC7942. Mol Microbiol 43:425–435. doi:10.1046/j.1365-2958.2002.02753.x. 11985719

[13] Dou Z, Heinhorst S, Williams EB, Murin CD, Shively JM, Cannon GC 2008 CO_2_ fixation kinetics of *Halothiobacillus neapolitanus* mutant carboxysomes lacking carbonic anhydrase suggest the shell acts as a diffusional barrier for CO_2_. J Biol Chem 283:10377–10384. doi:10.1074/jbc.M709285200. 18258595

[14] Price GD, Howitt SM, Harrison K, Badger MR 1993 Analysis of a genomic DNA region from the cyanobacterium *Synechococcus* sp. strain PCC7942 involved in carboxysome assembly and function. J Bacteriol 175:2871–2879. doi:10.1128/jb.175.10.2871-2879.1993. 8491708 PMC204604

[15] Cameron JC, Wilson SC, Bernstein SL, Kerfeld CA 2013 Biogenesis of a bacterial organelle: the carboxysome assembly pathway. Cell 155:1131–1140. doi:10.1016/j.cell.2013.10.044. 24267892

[16] Ryan P, Forrester TJB, Wroblewski C, Kenney TMG, Kitova EN, Klassen JS, Kimber MS 2019 The small RbcS-like domains of the β-carboxysome structural protein, CcmM, bind rubisco at a site distinct from that binding the RbcS subunit. J Biol Chem 294:2593–2603. doi:10.1074/jbc.RA118.006330. 30591587 PMC6393606

[17] Wang H, Yan X, Aigner H, Bracher A, Nguyen ND, Hee WY, Long BM, Price GD, Hartl FU, Hayer-Hartl M 2019 Rubisco condensate formation by CcmM in β-carboxysome biogenesis. Nature 566:131–135. doi:10.1038/s41586-019-0880-5. 30675061

[18] Cot SS-W, So A-C, Espie GS 2008 A multiprotein bicarbonate dehydration complex essential to carboxysome function in cyanobacteria. J Bacteriol 190:936–945. doi:10.1128/JB.01283-07. 17993516 PMC2223583

[19] Kinney JN, Salmeen A, Cai F, Kerfeld CA 2012 Elucidating essential role of conserved carboxysomal protein CcmN reveals common feature of bacterial microcompartment assembly. J Biol Chem 287:17729–17736. doi:10.1074/jbc.M112.355305. 22461622 PMC3366800

[20] Kerfeld CA, Sawaya MR, Tanaka S, Nguyen CV, Phillips M, Beeby M, Yeates TO 2005 Protein structures forming the shell of primitive bacterial organelles. Science 309:936–938. doi:10.1126/science.1113397. 16081736

[21] Rae BD, Long BM, Badger MR, Price GD 2012 Structural determinants of the outer shell of β-carboxysomes in *Synechococcus elongatus* PCC 7942: roles for CcmK2, K3-K4, CcmO, and CcmL. PLoS One 7:e43871. doi:10.1371/journal.pone.0043871. 22928045 PMC3425506

[22] Sommer M, Sutter M, Gupta S, Kirst H, Turmo A, Lechno-Yossef S, Burton RL, Saechao C, Sloan NB, Cheng X, Chan L-J, Petzold CJ, Fuentes-Cabrera M, Ralston CY, Kerfeld CA 2019 Heterohexamers formed by CcmK3 and CcmK4 increase the complexity of beta carboxysome shells. Plant Physiol 179:156–167. doi:10.1104/pp.18.01190. 30389783 PMC6324227

[23] Sommer M, Cai F, Melnicki M, Kerfeld CA 2017 β-Carboxysome bioinformatics: identification and evolution of new bacterial microcompartment protein gene classes and core locus constraints. J Exp Bot 68:3841–3855. doi:10.1093/jxb/erx115. 28419380 PMC5853843

[24] Cai F, Sutter M, Cameron JC, Stanley DN, Kinney JN, Kerfeld CA 2013 The structure of CcmP, a tandem bacterial microcompartment domain protein from the β-carboxysome, forms a subcompartment within a microcompartment. J Biol Chem 288:16055–16063. doi:10.1074/jbc.M113.456897. 23572529 PMC3668761

[25] Sutter M, Wilson SC, Deutsch S, Kerfeld CA 2013 Two new high-resolution crystal structures of carboxysome pentamer proteins reveal high structural conservation of CcmL orthologs among distantly related cyanobacterial species. Photosynth Res 118:9–16. doi:10.1007/s11120-013-9909-z. 23949415

[26] Tanaka S, Kerfeld CA, Sawaya MR, Cai F, Heinhorst S, Cannon GC, Yeates TO 2008 Atomic-level models of the bacterial carboxysome shell. Science 319:1083–1086. doi:10.1126/science.1151458. 18292340

[27] Hihara Y, Kamei A, Kanehisa M, Kaplan A, Ikeuchi M 2001 DNA microarray analysis of cyanobacterial gene expression during acclimation to high light. Plant Cell 13:793–806. doi:10.1105/tpc.13.4.793. 11283337 PMC135531

[28] McGinn PJ, Price GD, Maleszka R, Badger MR 2003 Inorganic carbon limitation and light control the expression of transcripts related to the CO_2_-concentrating mechanism in the cyanobacterium *Synechocystis* sp. strain PCC6803. Plant Physiol 132:218–229. doi:10.1104/pp.019349. 12746527 PMC166967

[29] Rohnke BA, Singh SP, Pattanaik B, Montgomery BL 2018 RcaE-dependent regulation of carboxysome structural proteins has a central role in environmental determination of carboxysome morphology and abundance in *Fremyella diplosiphon*. mSphere 3:e00617-17. doi:10.1128/mSphere.00617-17. PMC5784247 29404416

[30] Sun Y, Wollman AJM, Huang F, Leake MC, Liu L-N 2019 Single-organelle quantification reveals stoichiometric and structural variability of carboxysomes dependent on the environment. Plant Cell 31:1648–1664. doi:10.1105/tpc.18.00787. 31048338 PMC6635877

[31] Mangan NM, Brenner MP 2014 Systems analysis of the CO_2_ concentrating mechanism in cyanobacteria. Elife 2014:e02043. doi:10.7554/eLife.02043. PMC4027813 24842993

[32] Oakley CA, Hopkinson BM, Schmidt GW 2012 A modular system for the measurement of CO_2_ and O_2_ gas flux and photosynthetic electron transport in microalgae. Limnol Oceanogr Methods 10:968–977. doi:10.4319/lom.2012.10.968.

[33] Douchi D, Liang F, Cano M, Xiong W, Wang B, Maness P-C, Lindblad P, Yu J 2019 Membrane-inlet mass spectrometry enables a quantitative understanding of inorganic carbon uptake flux and carbon concentrating mechanisms in metabolically engineered cyanobacteria. Front Microbiol 10:1356. doi:10.3389/fmicb.2019.01356. 31293533 PMC6604854

[34] Ludwig M, Sultemeyer D, Price GD 2000 Isolation of *ccmKLMN* genes from the marine cyanobacterium, *Synechococcus* sp PCC7002 (*Cyanophyceae*) and evidence that CcmM is essential for carboxysome assembly. J Phycol 36:1109–1119. doi:10.1046/j.1529-8817.2000.00028.x.

[35] Price GD, Badger MR 1989 Isolation and characterization of high CO_2_-requiring-mutants of the cyanobacterium *Synechococcus* PCC7942. Plant Physiol 91:514–525. doi:10.1104/pp.91.2.514. 16667063 PMC1062031

[36] Sültemeyer D, Klughammer B, Ludwig M, Badger MR, Price GD 1997 Random insertional mutagenesis used in the generation of mutants of the marine cyanobacterium *Synechococcus* sp. strain PCC7002 with an impaired CO_2_ concentrating mechanism. Funct Plant Biol 24:317–327. doi:10.1071/PP96124.

[37] Campbell D 1996 Complementary chromatic adaptation alters photosynthetic strategies in the cyanobacterium *Calothrix*. Microbiology 142:1255–1263. doi:10.1099/13500872-142-5-1255. 33725794

[38] Badger MR, Andrews TJ 1982 Photosynthesis and inorganic carbon usage by the marine cyanobacterium, *Synechococcus* sp. Plant Physiol 70:517–523. doi:10.1104/pp.70.2.517. 16662526 PMC1067180

[39] Badger MR, Palmqvist K, Yu J-W 1994 Measurement of CO_2_ and HCO_3_^−^ fluxes in cyanobacteria and microalgae during steady-state photosynthesis. Physiol Plant 90:529–536. doi:10.1111/j.1399-3054.1994.tb08811.x.

[40] Farquhar GD, von Caemmerer S, Berry JA 1980 A biochemical model of photosynthetic CO_2_ assimilation in leaves of C_3_ species. Planta 149:78–90. doi:10.1007/BF00386231. 24306196

[41] Long SP, Bernacchi CJ 2003 Gas exchange measurements, what can they tell us about the underlying limitations to photosynthesis? Procedures and sources of error. J Exp Bot 54:2393–2401. doi:10.1093/jxb/erg262. 14512377

[42] Kakani VG, Surabhi GK, Reddy KR 2008 Photosynthesis and fluorescence responses of C_4_ plant *Andropogon gerardii* acclimated to temperature and carbon dioxide. Photosynthetica 46:420–430. doi:10.1007/s11099-008-0074-0.

[43] Pfeffer M, Peisker M 1998 CO_2_ gas exchange and phosphoenolpyruvate carboxylase activity in leaves of *Zea mays* L. Photosynth Res 58:281–291. doi:10.1023/A:1006188705423.

[44] Bennett A, Bogorad L 1973 Complementary chromatic adaptation in a filamentous blue-green alga. J Cell Biol 58:419–435. doi:10.1083/jcb.58.2.419. 4199659 PMC2109051

[45] Montgomery BL 2016 Mechanisms and fitness implications of photomorphogenesis during chromatic acclimation in cyanobacteria. J Exp Bot 67:4079–4090. doi:10.1093/jxb/erw206. 27217547

[46] Bordowitz JR, Montgomery BL 2008 Photoregulation of cellular morphology during complementary chromatic adaptation requires sensor-kinase-class protein RcaE in *Fremyella diplosiphon*. J Bacteriol 190:4069–4074. doi:10.1128/JB.00018-08. 18390655 PMC2395048

[47] Kehoe DM, Grossman AR 1996 Similarity of a chromatic adaptation sensor to phytochrome and ethylene receptors. Science 273:1409–1412. doi:10.1126/science.273.5280.1409. 8703080

[48] Singh SP, Montgomery BL 2014 Morphogenes *bolA* and *mreB* mediate the photoregulation of cellular morphology during complementary chromatic acclimation in *Fremyella diplosiphon*. Mol Microbiol 93:167–182. doi:10.1111/mmi.12649. 24823920

[49] Terauchi K, Montgomery BL, Grossman AR, Lagarias JC, Kehoe DM 2004 RcaE is a complementary chromatic adaptation photoreceptor required for green and red light responsiveness. Mol Microbiol 51:567–577. doi:10.1046/j.1365-2958.2003.03853.x. 14756794

[50] Brooks A, Farquhar GD 1985 Effect of temperature on the CO_2_/O_2_ specificity of ribulose-1,5-bisphosphate carboxylase/oxygenase and the rate of respiration in the light. Planta 165:397–406. doi:10.1007/BF00392238. 24241146

[51] Gutu A, Kehoe DM 2012 Emerging perspectives on the mechanisms, regulation, and distribution of light color acclimation in cyanobacteria. Mol Plant 5:1–13. doi:10.1093/mp/ssr054. 21772031

[52] Graan T, Ort DR 1986 Detection of oxygen-evolving photosystem II centers inactive in plastoquinone reduction. Biochim Biophys Acta Bioenerg 852:320–330. doi:10.1016/0005-2728(86)90238-0.

[53] Mulo P, Laakso S, Mäenpää P, Aro E-M 1998 Stepwise photoinhibition of photosystem II. Plant Physiol 117:483–490. doi:10.1104/pp.117.2.483. 9625701 PMC34968

[54] Lechno-Yossef S, Rohnke BA, Belza ACO, Melnicki MR, Montgomery BL, Kerfeld CA 2020 Cyanobacterial carboxysomes contain an unique rubisco-activase-like protein. New Phytol 225:793–806. doi:10.1111/nph.16195. 31518434

[55] Montgomery BL, Lechno-Yossef S, Kerfeld CA 2016 Interrelated modules in cyanobacterial photosynthesis: the carbon-concentrating mechanism, photorespiration, and light perception. J Exp Bot 67:2931–2940. doi:10.1093/jxb/erw162. 27117337

[56] Omata T, Gohta S, Takahashi Y, Harano Y, Maeda S 2001 Involvement of a CbbR homolog in low CO_2_-induced activation of the bicarbonate transporter operon in cyanobacteria. J Bacteriol 183:1891–1898. doi:10.1128/JB.183.6.1891-1898.2001. 11222586 PMC95083

[57] Eisenhut M, von Wobeser EA, Jonas L, Schubert H, Ibelings BW, Bauwe H, Matthijs HCP, Hagemann M 2007 Long-term response toward inorganic carbon limitation in wild type and glycolate turnover mutants of the cyanobacterium *Synechocystis* sp. strain PCC 6803. Plant Physiol 144:1946–1959. doi:10.1104/pp.107.103341. 17600135 PMC1949882

[58] Schwarz D, Nodop A, Hüge J, Purfürst S, Forchhammer K, Michel K-P, Bauwe H, Kopka J, Hagemann M 2011 Metabolic and transcriptomic phenotyping of inorganic carbon acclimation in the cyanobacterium *Synechococcus elongatus* PCC 7942. Plant Physiol 155:1640–1655. doi:10.1104/pp.110.170225. 21282404 PMC3091134

[59] McKay RML, Gibbs SP, Espie GS 1993 Effect of dissolved inorganic carbon on the expression of carboxysomes, localization of Rubisco and the mode of inorganic carbon transport in cells of the cyanobacterium Synechococcus UTEX 625. Arch Microbiol 159:21–29. doi:10.1007/BF00244259.

[60] Cobley JG, Zerweck E, Reyes R, Mody A, Seludo-Unson JR, Jaeger H, Weerasuriya S, Navankasattusas S 1993 Construction of shuttle plasmids which can be efficiently mobilized from *Escherichia coli* into the chromatically adapting cyanobacterium, *Fremyella diplosiphon*. Plasmid 30:90–105. doi:10.1006/plas.1993.1037. 8234495

[61] Bustin SA, Benes V, Garson JA, Hellemans J, Huggett J, Kubista M, Mueller R, Nolan T, Pfaffl MW, Shipley GL, Vandesompele J, Wittwer CT 2009 The MIQE guidelines: minimum information for publication of quantitative real-time PCR experiments. Clin Chem 55:611–622. doi:10.1373/clinchem.2008.112797. 19246619

[62] de Marsac NT, Houmard J 1988 Complementary chromatic adaptation: physiological conditions and action spectra, p 318–328. *In* Methods in enzymology. Academic Press, San Diego, CA. doi:10.1016/0076-6879(88)67037-6.

[63] Singh SP, Montgomery BL 2011 Temporal responses of wild-type pigmentation and RcaE-deficient strains of *Fremyella diplosiphon* during light transitions. Commun Integr Biol 4:503–510. doi:10.4161/cib.16788. 22046449 PMC3204114

