# Supplementary figures and images for: Linking the Dynamic Response of the Carbon Dioxide-Concentrating Mechanism to Carbon Assimilation Behavior in Fremyella diplosiphon
Source: mBio. 2020 May 26;11(3):e01052-20. doi: 10.1128/mBio.01052-20 (PMC7251215; doi:10.1128/mBio.01052-20)

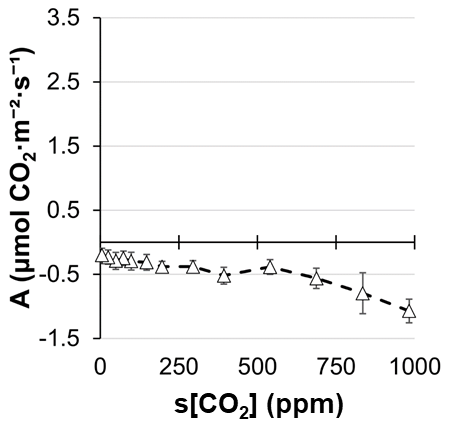

Supplement: FIG S1 [file mBio.01052-20-sf001.tif]

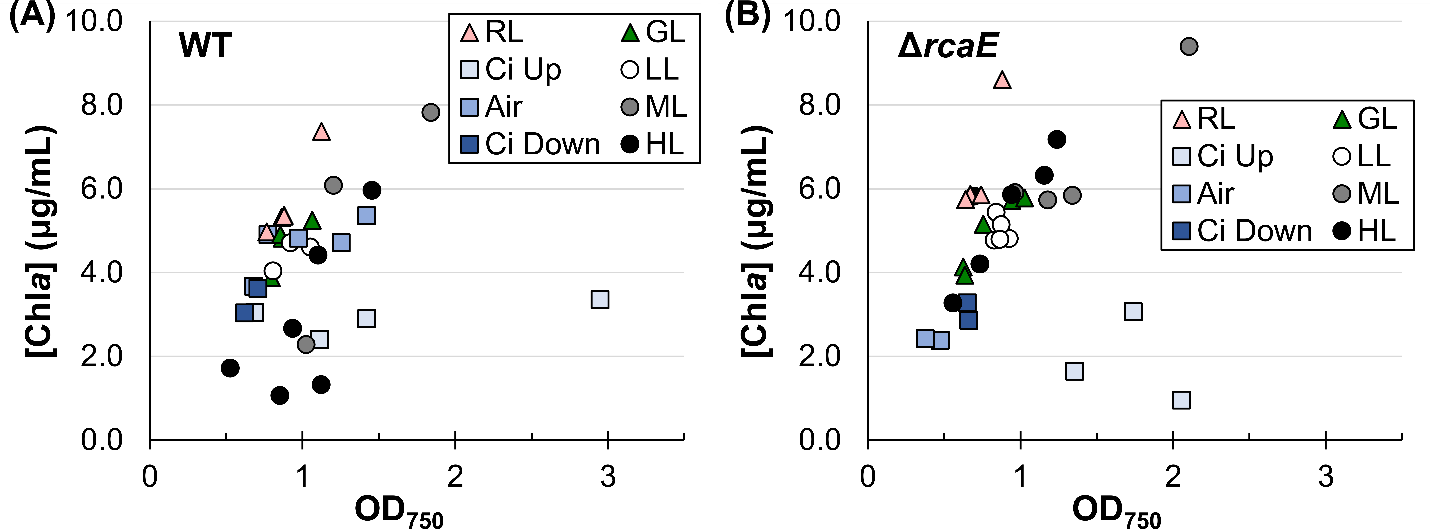

Supplement: FIG S2 [file mBio.01052-20-sf002.tif]

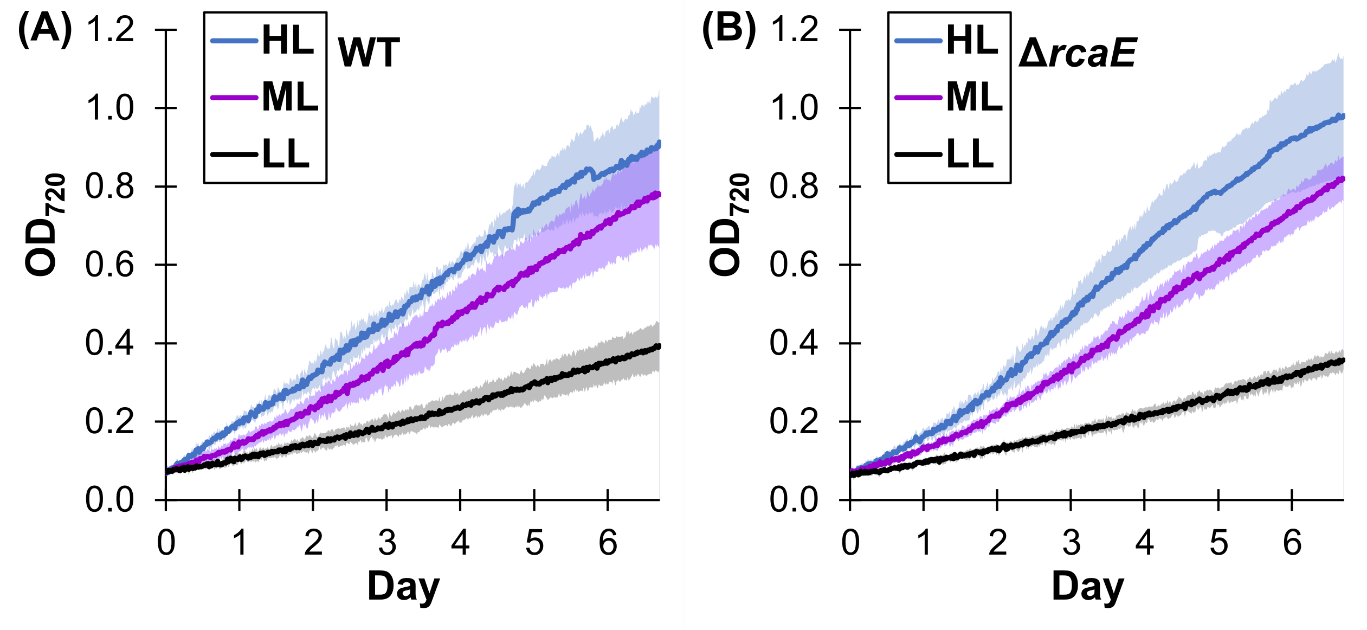

Supplement: FIG S3 [file mBio.01052-20-sf003.tif]

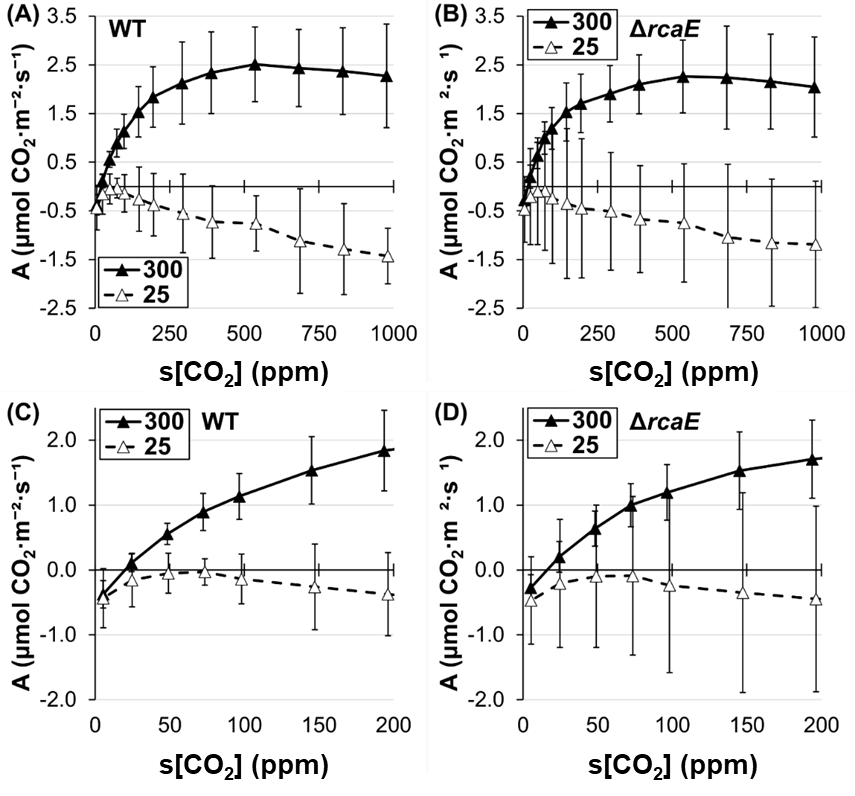

Supplement: FIG S4 [file mBio.01052-20-sf004.tif]
